# CBFA2T3-GLIS2 model of pediatric acute megakaryoblastic leukemia identifies FOLR1 as a CAR T cell target

**DOI:** 10.1172/JCI157101

**Published:** 2022-11-15

**Authors:** Quy Le, Brandon Hadland, Jenny L. Smith, Amanda Leonti, Benjamin J. Huang, Rhonda Ries, Tiffany A. Hylkema, Sommer Castro, Thao T. Tang, Cyd N. McKay, LaKeisha Perkins, Laura Pardo, Jay Sarthy, Amy K. Beckman, Robin Williams, Rhonda Idemmili, Scott Furlan, Takashi Ishida, Lindsey Call, Shivani Srivastava, Anisha M. Loeb, Filippo Milano, Suzan Imren, Shelli M. Morris, Fiona Pakiam, Jim M. Olson, Michael R. Loken, Lisa Brodersen, Stanley R. Riddell, Katherine Tarlock, Irwin D. Bernstein, Keith R. Loeb, Soheil Meshinchi

**Affiliations:** 1 Fred Hutchinson Cancer Research Center, Seattle, Washington, USA.; 2 Department of Pediatrics, University of Washington, Seattle, Washington, USA.; 3 Department of Pediatrics and; 4 Helen Diller Family Comprehensive Cancer Center, University of California San Francisco, San Francisco, California, USA.; 5 Department of Laboratory Medicine & Pathology, University of Minnesota, Minneapolis, Minnesota, USA.; 6 Seattle Children’s Research Institute, Seattle, Washington, USA.; 7 Hematologics, Inc, Seattle, Washington, USA.; 8 Department of Laboratory Medicine and Pathology, University of Washington, Seattle, Washington, USA.; 9 Children’s Oncology Group, Monrovia, California, USA.

**Keywords:** Oncology, Therapeutics, Cancer immunotherapy, Leukemias, Oncogenes

## Abstract

The CBFA2T3-GLIS2 (C/G) fusion is a product of a cryptic translocation primarily seen in infants and early childhood and is associated with dismal outcome. Here, we demonstrate that the expression of the C/G oncogenic fusion protein promotes the transformation of human cord blood hematopoietic stem and progenitor cells (CB HSPCs) in an endothelial cell coculture system that recapitulates the transcriptome, morphology, and immunophenotype of C/G acute myeloid leukemia (AML) and induces highly aggressive leukemia in xenograft models. Interrogating the transcriptome of C/G-CB cells and primary C/G AML identified a library of C/G-fusion-specific genes that are potential targets for therapy. We developed chimeric antigen receptor (CAR) T cells directed against one of the targets, folate receptor α (FOLR1), and demonstrated their preclinical efficacy against C/G AML using in vitro and xenograft models. FOLR1 is also expressed in renal and pulmonary epithelium, raising concerns for toxicity that must be addressed for the clinical application of this therapy. Our findings underscore the role of the endothelial niche in promoting leukemic transformation of C/G-transduced CB HSPCs. Furthermore, this work has broad implications for studies of leukemogenesis applicable to a variety of oncogenic fusion-driven pediatric leukemias, providing a robust and tractable model system to characterize the molecular mechanisms of leukemogenesis and identify biomarkers for disease diagnosis and targets for therapy.

## Graphical abstract

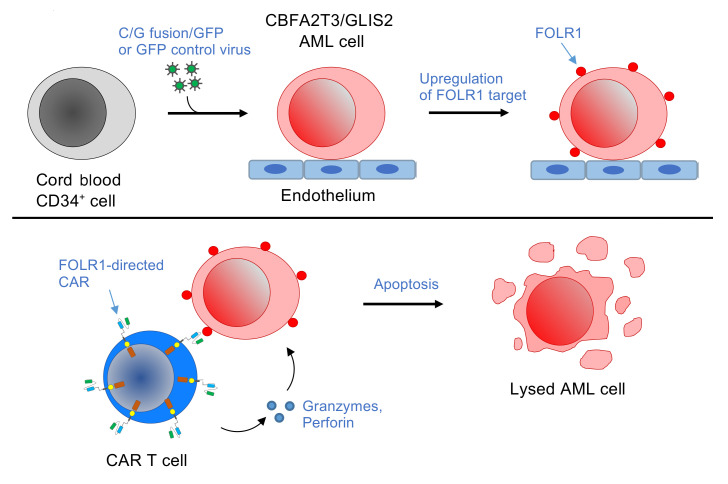

## Introduction

Most pediatric acute myeloid leukemias (AMLs) are driven by the expression of oncogenic fusion proteins that in some cases are predicted to be sufficient for leukemogenesis (1). Among these, CBFA2T3-GLIS2 (C/G) fusion is a product of a cryptic translocation in non–Down syndrome acute megakaryoblastic leukemia (AMKL) (2). CBFA2T3 (also called ETO2) is a member of the ETO family of transcription factors that was initially identified as a RUNX1 fusion partner in adult therapy-related AML (3). GLIS2 is a zinc finger transcription factor regulated by the Hedgehog pathway (4, 5). The C/G fusion protein binds to DNA via CBFA2T3-associated transcription factors or directly through GLIS2 zinc fingers, altering the expression of key regulatory factors, including upregulation of the ETS transcription factor ERG leading to enhanced self-renewal and downregulation of GATA1, inhibiting differentiation. C/G AML accounts for approximately 5% of all pediatric AMLs and is primarily found in infants as AMKL and AML in early childhood (2, 6–9). The presence of the fusion is associated with a dismal prognosis, with overall survival rates ranging from 15% to 30% (10). Since C/G AMLs possess a cryptic translocation that is often missed in cytogenetic studies, the clinical identification of C/G AML is dependent on the unique immunophenotype of high CD56 expression with dim/negative expression of CD45 and CD38, termed the RAM immunophenotype (11).

Similar to other AMKL fusions that promote highly aggressive disease in children, the C/G fusion is rarely associated with additional recurrent mutations (1, 2, 8, 9), suggesting that the fusion might be sufficient for malignant transformation. Initial studies expressing the C/G fusion in murine bone marrow cells showed that the fusion induces abnormal megakaryocytic differentiation and increased self-renewal but lacks the capacity to induce overt leukemia in recipient mice (2, 12, 13). Using an inducible C/G mouse model, Lopez et al. recently demonstrated that the fusion is sufficient for leukemic transformation when expressed in hematopoietic stem and progenitor cells (HPSCs), where the developmental stage of the HPSCs dictates the disease phenotype (i.e., transformed fetal liver–stage HSPCs produce AMKL, whereas adult bone marrow HSPCs generate AML) (14). These results may explain why infants with C/G develop AMKL, whereas older children with this fusion develop AML (14). To generate a human model of C/G AMKL, we previously transduced human cord blood (CB) CD34^+^ HSPCs with a C/G expression construct and observed partial recapitulation of the AMKL phenotype, including enhanced proliferation and abnormal megakaryocytic differentiation in culture, and the RAM immunophenotype (8).

The tumor microenvironment and extracellular signals play an important role during leukemogenesis. The vascular niche endothelial cells (ECs) have been shown to be important for normal and malignant hematopoiesis, contributing to maintenance and self-renewal of HSPCs as well as supporting leukemic progression, leukemia precursor survival, and drug resistance (15–18). Previous studies have demonstrated that human umbilical vein ECs (HUVECs) transduced with E4ORF1 virus (E4 ECs) support the expansion of CB HSPCs (19) and provide efficient conditions for long-term culture of primary AML precursors (17), thus effectively recapitulating the EC niche ex vivo. In this study, we established an innovative EC coculture system using E4 ECs to provide the microenvironmental support necessary to promote leukemic transformation and self-renewal ex vivo. Using this system, we established long-term cultures of C/G-transformed cells that closely resemble human AMKL. We further demonstrated the utility of this EC coculture model to characterize C/G AMKL transformation and identify therapeutic targets.

## Results

### C/G expression is sufficient to transform human CB HSPCs and induces leukemia in mouse xenografts.

**Figure 1:**
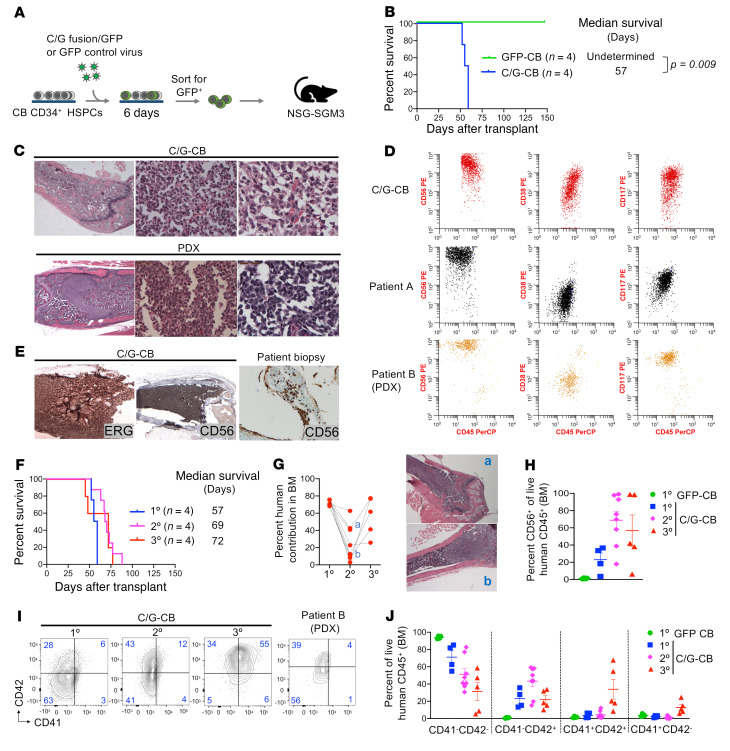
C/G-CB cells induce leukemia, recapitulating primary disease. (**A**) Diagram of experimental design. (**B**) Kaplan-Meier survival curves of NSG-SGM3 mice transplanted with GFP-CB control and C/G-CB cells. Statistical differences in survival were evaluated using the Mantel-Cox log-rank test. *n* = 4 mice per group. (**C**) Representative histology of H&E stain of femur taken from a mouse transplanted with C/G-CB cells (top) and cells from a C/G-positive patient sample (bottom) after development of leukemia. Original magnification, ×2.5 (left), ×40 (middle), and ×63 (right). See Supplemental Figure 1 for all H&E stains from C/G-CB–transplanted mice. *n* = 4 mice for C/G-CB cells and *n* = 2 mice for PDX. (**D**) Expression of the RAM immunophenotype in C/G-CB cells harvested from the bone marrow (BM) of a representative mouse at necropsy compared to a primary patient sample and PDX marrow xenograft cells. In all 3 samples, malignant cells were gated based on human CD45 expression and SSC. *n* = 4 mice for C/G-CB cells, *n* = 2 mice for PDX derived from BM cells of patient B. (**E**) Left and middle: Representative immunohistochemistry showing high expression of ERG (×10 magnification) and CD56 (×5 magnification) in the femur of a representative mouse transplanted with C/G-CB cells. Right: Small aggregates of blasts with high CD56 expression detected in a BM biopsy of a chemotherapy-refractory C/G-fusion-positive patient, consistent with residual, adherent, patchy disease distribution (×100 magnification). *n* = 4 mice. (**F**) Kaplan-Meier plot showing survival in primary (1°, *n* = 4 mice), secondary (2°, *n* = 7 mice), and tertiary (3°, *n* = 5 mice) transplantations of C/G-CB cells. (**G**) Engraftment of C/G-CB cells in the BM at time of symptomatic leukemia, shown as percentage human CD45^+^ cells. Images on the right are H&E stain of femurs taken from mice indicated by “a” and “b.” See Supplemental Figure 1 for all H&E stains from C/G-CB–transplanted mice. (**H**) Quantification of CD56^+^ cells among human CD45^+^ cells isolated from the BM at necropsy following development of symptomatic leukemia. (**I**) Expression of AMKL markers, CD41 and CD42, in C/G-CB and PDX cells harvested from the BM at necropsy. C/G-CB cells were gated on human CD45^+^ cells. PDX cells were gated on human CD45^+^CD56^+^ cells. (**J**) Quantification of CD41/CD42 subsets described in **I**. Bars indicate mean ± SEM. (**G**–**J**) 1°, *n* = 4 mice per group; 2°, *n* = 7 mice; and 3°, *n* = 5 mice.

Given the importance of the developmental context in which the C/G fusion can induce AMKL, we expressed the C/G fusion or GFP control in human CB HSPCs (C/G-CB or GFP-CB) by lentiviral transduction and transplanted the transduced cells into NSG-SGM3 mice (Figure 1A). Within 60 days of transplantation, all mice (4 of 4) injected with C/G-CB cells developed florid leukemia, while all control mice (4 of 4) survived until the study endpoint (Figure 1B). Histology of the femur from C/G-CB–xenograft mice revealed extensive leukemia with bone remodeling resembling the pathology observed in xenograft mice bearing C/G patient–derived leukemia cells (PDX; Figure 1C and Supplemental Figure 1; supplemental material available online with this article; https://doi.org/10.1172/JCI157101DS1). The leukemic cells had a unique appearance, with focal intercellular adhesions in the less dense areas to sheets of cohesive cells in the more cellular areas. Flow cytometric analysis of marrow C/G-CB–xenograft cells identified a malignant population with a RAM immunophenotype (CD56^hi^, CD45^dim^, and CD38^dim/–^; Figure 1D) previously reported in infants with C/G AML (11, 20). Immunohistochemistry further showed high expression of ERG and CD56 (markers associated with C/G AML; refs. 11, 13, 20) in the mouse bone marrow, characteristic of this type of leukemia, and similar to the high CD56 expression in leukemia aggregates present in a bone marrow biopsy from a C/G patient (Figure 1E).

To evaluate whether C/G imparts enhanced self-renewal to leukemia-initiating cells (LICs), we performed serial transplantation of C/G-CB cells. All mice from secondary (8 of 8) and tertiary (5 of 5) transplants also developed AML, with a median survival of 69 and 72 days, respectively (Figure 1F). Bone marrow engraftment of C/G-CB cells as measured by flow cytometry was variable in these mice at time of symptomatic disease (5%–70%; Figure 1G), ranging from prominent leukemia with replacement of the normal hematopoiesis to small focal clusters of leukemia cells (Supplemental Figure 1). For example, mouse A had 40% engraftment by flow cytometry and extensive leukemia by morphology (Figure 1G), while mouse B had only 9% engraftment by flow cytometry with focal tumor nodules near the metaphysis and distal epiphysis of the femur as detected by morphology (Figure 1G). Serial transplantation of the C/G-CB cells from both mice produced leukemia in tertiary recipients. The unique cellular features with intercellular adhesion and focal cohesive tumors demonstrate the adherent nature of C/G-CB cells (Figure 1E).

Flow cytometric analysis of C/G-CB–xenograft cells revealed immunophenotypic evolution during the serial transplantations, with an expanded population of CD56^+^ cells in the bone marrow (Figure 1H) and in extramedullary disease (Supplemental Figure 2, A and B). AMKL is a form of AML that is characterized by immature blasts expressing the megakaryocytic marker CD41, CD42, or CD61 (21). Immunophenotype analysis of C/G-CB cells revealed an aberrant megakaryocytic subset (CD41^–^CD42^+^) in the primary and subsequent serial transplantations (Figure 1I and Supplemental Figure 2C). Bertuccio et al. previously identified a similar subpopulation of CD41^–^CD42^+^ cells with a gene expression profile most closely matching that of human C/G leukemia (22). Furthermore, this aberrant megakaryocytic subset was also detected in primary C/G AML cells from the PDX model (Figure 1I). Monitoring CD41 and CD42 expression during serial transplantation showed an immunophenotypic evolution from CD41^–^CD42^+^ to CD41^+^CD42^+^ subsets (Figure 1, I and J, and Supplemental Figure 2C). Taken together, these results demonstrate that the expression of C/G induces transformation of CB HSPCs that resembles the morphology and immunophenotype of human C/G AMKL in xenograft models.

### Culture of C/G-transduced CD34^+^ CB cells with ECs promotes long-term self-renewal and leukemic transformation.

**Figure 2:**
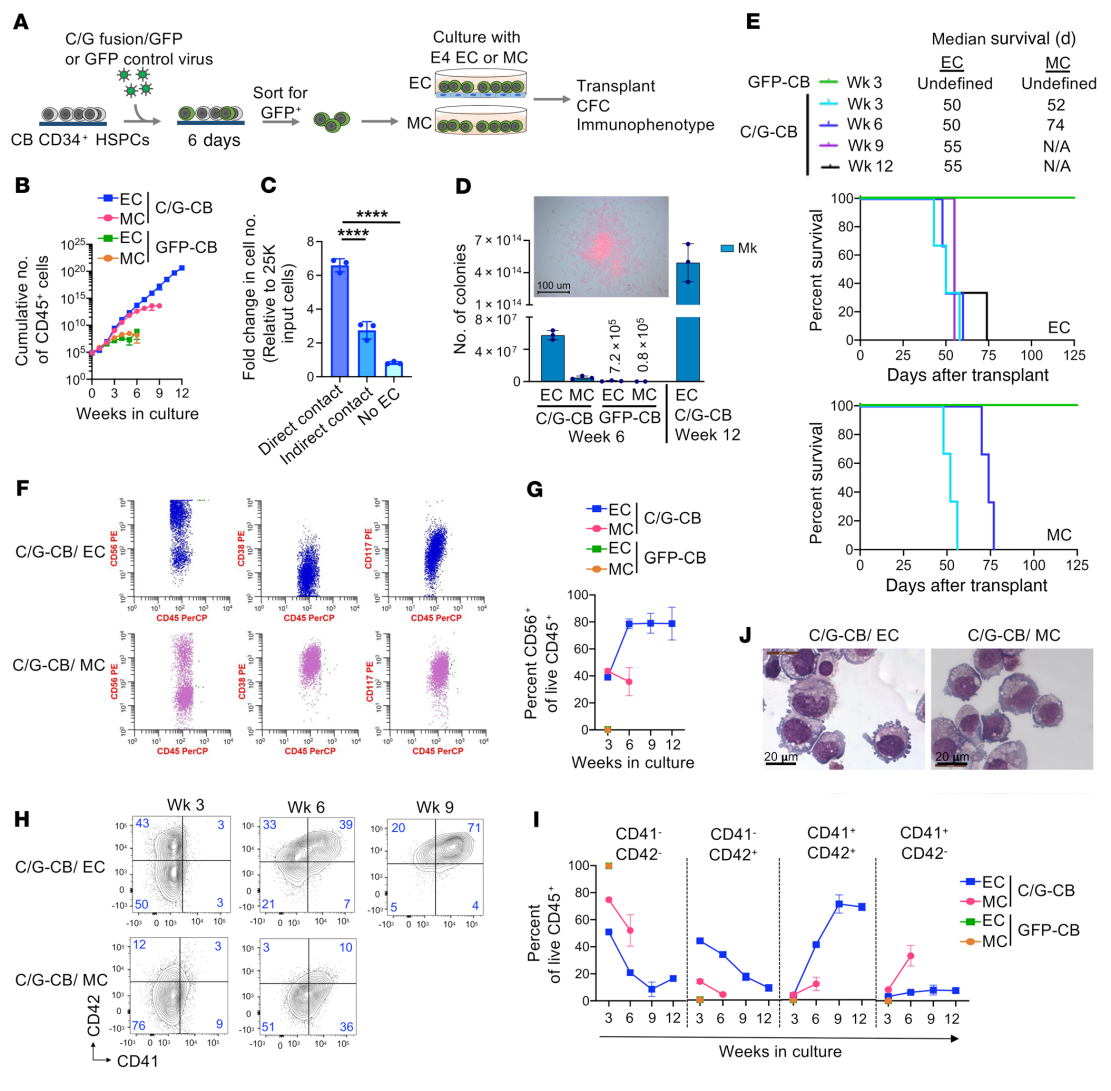
ECs enhance the proliferative potential and promote leukemic progression of C/G-CB cells. (**A**) Diagram of experimental design. This experiment was performed twice using 2 separate CB units. See Supplemental Figure 4 for the repeat experiment. (**B**) Growth kinetics of C/G-CB and GFP-CB cells in EC coculture or MC. (**C**) C/G-CB cells expanded in EC coculture for 9 weeks were reseeded in EC coculture either directly (direct contact) or in EC Transwells (indirect contact) or placed in liquid culture containing SFEM II (with SCF, FLT3L, and TPO). After 7 days, the number of GFP^+^ cells was quantified by flow cytometry. Data in **B** and **C** presented as mean ± SD from 3 technical replicates. Statistical significance was determined by 1-way ANOVA. ****P < 0.00005. (**D**) At 6 and 12 weeks, a fraction of each culture was transferred to MegaCult cultures. Colonies derived from megakaryocytic (Mk) progenitors were scored and enumerated. Data were normalized to the 500 input cells at the start of the EC coculture or MC culture. A representative colony stained with anti–human CD41 and an alkaline phosphatase detection system is shown. Data shown are the average of 3 technical replicates. Error bars denote SD. (**E**) Equivalent number of C/G-CB and GFP-CB cells in EC coculture or MC were transplanted into NSG-SGM3 mice at indicated time points (5 × 10^6^/mouse at week 3, and 1 × 10^7^/mouse at weeks 6, 9, and 12). Due to insufficient expansion, GFP-CB cells were not transplanted after 3 weeks in either condition, similarly for C/G-CB cells after 6 weeks in MC culture. Median survival and Kaplan-Meier survival curve are shown. C/G-CB (*n* = 3 mice/group), GFP-CB (*n* = 2 mice/group). (**F**) Expression of the RAM immunophenotype in C/G-CB cells after 6 weeks in EC coculture or MC. Data are pooled from 3 technical replicates. (**G**) Quantification of CD56^+^ cells among live CD45^+^ cells over weeks in culture. (**H** and **I**) Expression of CD41 and CD42 (**H**) and quantification of CD41/CD42 subsets (**I**) at indicated time points in EC coculture or MC. Data in **H** and **I** presented as mean ± SD from 3 technical replicates. (**J**) Morphological evaluation of the C/G-CB cells cultured with ECs or in MC for 9 weeks showed features of megakaryocytic differentiation, including open chromatin, prominent nucleoli, and abundant focal, basophilic, and vacuolated cytoplasm with cytoplasmic blebbing. Results shown are representative of 3 technical replicates. Scale bars: 100 μm (**D**) and 20 μm (**J**).

We next designed a long-term culture system using E4 ECs to support the growth and transformation of transduced CB stem cells to model and monitor the changes that occur during leukemogenesis. To assess whether E4 ECs support leukemic transformation of C/G fusion, we cultured C/G-CB cells in E4 EC coculture (19) and compared them to cells grown in myeloid-promoting conditions (MC) that we used previously (Figure 2A and ref. 23). C/G-CB cells expanded faster, with prolonged lifespan in EC coculture compared with MC, as determined by the cumulative number of GFP^+^ cells (Figure 2B). In contrast, control GFP-CB cells exhibited limited, short-lived proliferation that ended after 3 weeks in either condition. The enhanced proliferation of C/G-CB cells required continuous and direct exposure to the ECs, since transfer to either an EC Transwell culture or culture without ECs (Figure 2C) resulted in decreased proliferation, suggesting that the growth-promoting effect of the ECs is mediated by direct contact in addition to secreted factors.

The C/G fusion has been previously shown to confer self-renewal to hematopoietic progenitors (2, 13) that was enhanced in the long-term EC cocultures. At 6 weeks, C/G-CB cells in EC coculture formed significantly more megakaryocytic colonies than C/G-CB cells grown in MC or control GFP cells grown in either condition. Strikingly, even after 12 weeks, C/G-CB cells cultured in EC coculture produced a large number of megakaryocytic colonies (Figure 2D), demonstrating long-lived self-renewal of the C/G-CB cells cocultured with ECs. To determine whether the EC niche promotes the generation and propagation of leukemia-generating cells, we transplanted C/G-CB cells expanded on ECs or in MC following 3, 6, 9, and 12 weeks of culture into NSG-SGM3 mice. Remarkably, C/G-CB cells from each time point of EC coculture exhibited robust engraftment that progressed to frank leukemia in vivo (Figure 2E and Supplemental Figure 3), demonstrating that EC coculture promotes long-term maintenance of functional LICs. In contrast, C/G-CB cells grown in the MC exhibited limited growth in culture (>6 weeks), with cells from 3- and 6-week cultures able to induce leukemia in xenograft mice, suggesting limited preservation of the leukemia-generating cells.

To monitor leukemic immunophenotypic evolution during long-term EC cultures, we assessed the expression of cell surface proteins, focusing on AMKL markers and the RAM immunophenotype characteristic of C/G AMKL. Brodersen et al. previously reported an immunophenotype diagnostic of C/G AMKL cells, the RAM immunophenotype, characterized by high CD56 expression and dim/negative CD45 and CD38 expression that is diagnostic of C/G AMKL (11). After 6 weeks, the C/G-CB cells grown in EC coculture exhibited a near homogeneous immunophenotype consistent with the RAM immunophenotype, while only a small subset of RAM-positive cells was evident following growth in the MC (Figure 2F). The high percentage of CD56^+^ cells was maintained in the EC coculture for more than 6 weeks (Figure 2G). Cells grown in the 2 culture conditions also exhibited distinct patterns of expression of megakaryocytic markers CD41 and CD42. Cells grown in the EC coculture exhibited abnormal maturation, with an aberrant CD41^–^CD42^+^ population that developed by week 3 and then progressed to the CD41^+^CD42^+^ phenotype with a subpopulation of CD41^–^CD42^+^ cells (Figure 2, H and I). An abnormal CD41^–^CD42^+^ population has been identified in other studies and shown to express a stem cell signature (14). In contrast, only a small population of CD41^+^CD42^+^ cells was detected in the MC culture after 6 weeks. Morphological evaluation showed megakaryocytic features among C/G-CB cells in both culture conditions (Figure 2J), consistent with the immunophenotype. These results were reproduced in a separate experiment with CB HSPCs from another donor (Supplemental Figure 4). Thus, EC coculture supports the development of C/G-transformed CB HSPCs, with an evolution to leukemia with the expression of the RAM immunophenotype that closely resembles primary C/G AML.

### ECs promote malignant transformation in C/G-CB cells that correlate transcriptionally with human disease.

**Figure 3:**
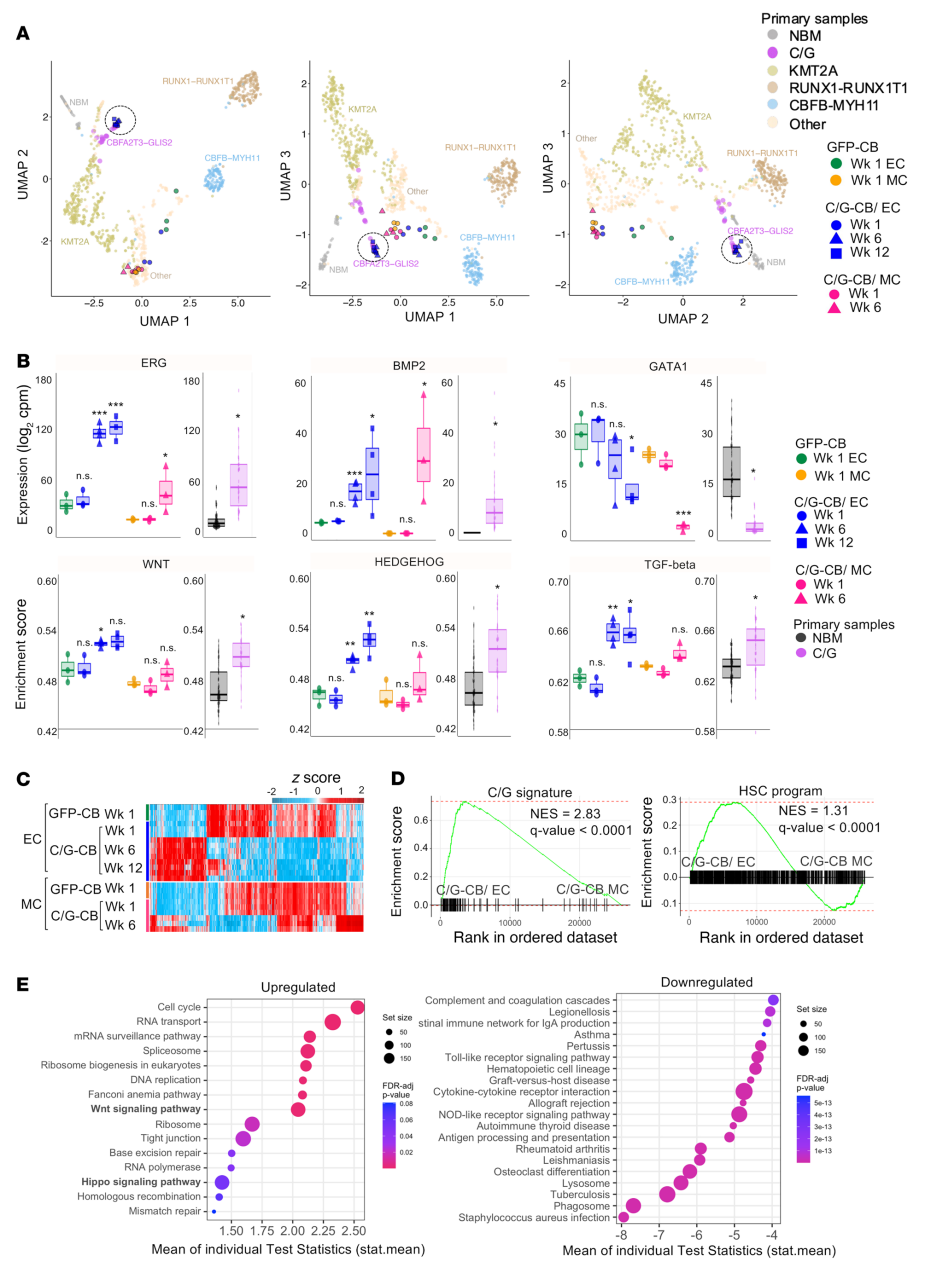
Transcriptional profile of C/G-CB cells in EC coculture recapitulates primary C/G AML. (**A**) Unsupervised clustering by uniform manifold and projection (UMAP) analysis of C/G-CB and GFP-CB cells in reference to primary AML samples. Dashed circle indicates C/G-CB cells cocultured with ECs at week 6 and 12 time points. Normal bone marrow (NBM, *n* = 68); KMT2A (*n* = 319); RUNX1-RUNX1T1 (*n* = 157); CBFB-MYH11 (*n* = 120); other (*n* = 444); CBFA2T3-GLIS2 (*n* = 39). Primary patient data are described in Smith et al. (8). For cultured cells, *n* = 4 technical replicates for C/G-CB cells in EC coculture at week; *n* = 3 technical replicates for all other groups. (**B**) Top: Expression of ERG, BMP2, and GATA1 in GFP-CB versus C/G-CB cells over weeks in EC and MC conditions as well as in C/G-fusion-positive primary versus NBM samples. Bottom: Single-sample gene set enrichment (ssGSEA) scores of Hedgehog, TGF-β, and WNT signaling pathways for GFP-CB versus C/G-CB cells and NBM samples versus primary-fusion-positive samples. CBFA2T3-GLIS2 primary samples (*n* = 39); NBM samples (*n* = 68). For cultured cells, *n* = 4 technical replicates for C/G-CB cells in EC coculture at week; *n* = 3 technical replicates for all other groups. *P ≤ 0.05, **P ≤ 0.01, ***P ≤ 0.001 by unpaired, 2-sided, nonparametric Wilcoxon’s rank-sum test, analyzing differences in expression between C/G-CB in EC or MC conditions and GFP controls in EC or MC conditions, and differences between primary C/G AML samples and healthy NBM. (**C**) Heatmap of differentially expressed genes in C/G-CB versus GFP-CB cells in EC coculture or MC. (**D**) GSEA plots of C/G and HSC signature genes comparing C/G-CB cells in EC coculture versus MC at week 6 of culture. (**E**) Pathways that are upregulated (left) and downregulated (right) in C/G-CB cells in EC coculture compared with MC. (**C**–**E**) *n* = 4 technical replicates for C/G-CB cells in EC coculture at week 6; *n* = 3 technical replicates for all other groups.

To determine the fidelity of the C/G-transformed cells to primary leukemia, we evaluated gene expression by RNA sequencing (RNA-seq) of C/G-CB cells cultured with ECs or in MC. Remarkably, unsupervised clustering analysis demonstrated that the C/G-CB cells from weeks 6 and 12 in EC coculture clustered with primary C/G-positive patient samples, but separately from C/G-CB cells cultured in MC and GFP controls (Figure 3A). This suggested that the transcriptional signatures of primary C/G AML are similar to the C/G-CB cells cocultured with ECs. Further transcriptome analysis revealed overexpression of *ERG* and *BMP2*, downstream genes previously shown to be strongly upregulated in C/G AML (2, 13), and decreased expression of the erythroid-megakaryocyte differentiation gene *GATA1* (24–28), downregulated in C/G AML (13), in both EC coculture and MC (Figure 3B). However, there were significant differences in the global expression profiles of C/G-CB cells from EC coculture compared with MC (Figure 3C), suggesting differences in evolution to leukemia in the 2 culture conditions.

To determine the effects of ECs on malignant transformation, we assessed the status of the WNT, HEDGEHOG, and TGF-β pathways known to be dysregulated in C/G leukemia (2, 8). These pathways were highly enriched in C/G-CB cells grown in EC coculture but not in MC (Figure 3B). We previously demonstrated that a number of cell adhesion molecules and integrins are upregulated in C/G leukemia (8). A majority of these genes were upregulated in C/G-CB cells independent of the culture condition (Supplemental Figure 5A), suggesting that dysregulated expression of these genes is determined by the fusion and not the microenvironment. The expression of cell adhesion molecules and integrins presumably contributes to the focal distribution and adherent morphology identified in the C/G-CB–xenograft mice (Figure 1C and Supplemental Figure 1).

Gene set enrichment analysis (GSEA) further revealed that C/G and hematopoietic stem cell (HSC) signature genes, previously shown to be associated with C/G AML (8, 13), were both significantly enriched in C/G-CB cells grown in EC culture relative to MC (Figure 3D and Supplemental Figure 5, B and C). Other C/G-specific pathways (see ref. 8) significantly enriched in the C/G-CB cells in EC coculture compared with MC were the Hippo signaling pathway and tight junction (Figure 3E). Together, these results suggest that ECs synergize with the C/G fusion to induce transcriptional programs that recapitulate primary C/G-driven AMKL.

### Malignant transformation is associated with upregulation of therapeutic target FOLR1.

**Figure 4:**
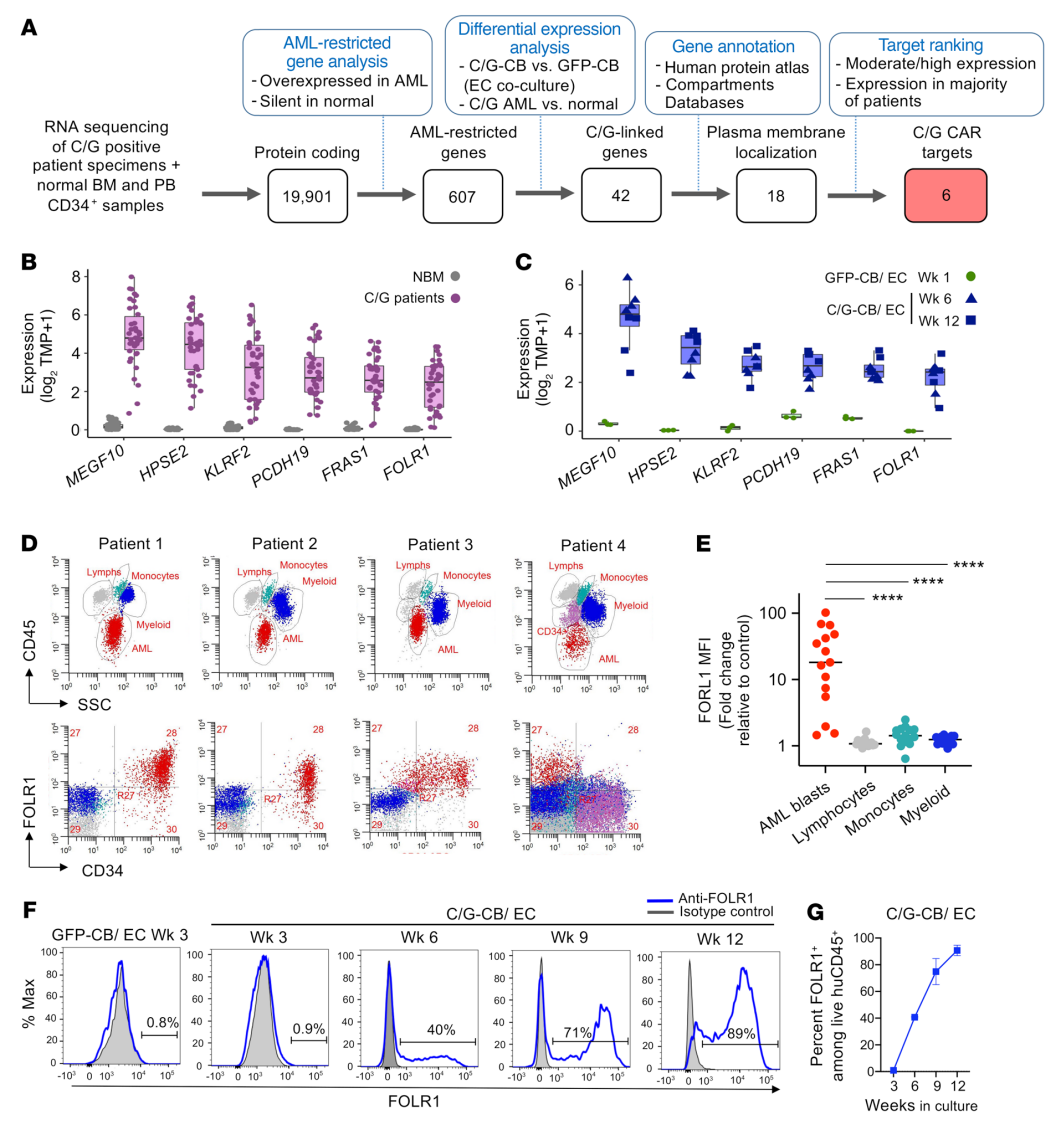
Integrative transcriptomics of primary samples and C/G-CB identify FOLR1 therapeutic target. (**A**) Diagram of computational workflow to identify C/G-specific CAR targets. See Methods and Supplemental Figure 6 for details. Normal tissues include bulk bone marrow (BM) samples and peripheral blood (PB) CD34^+^ samples. (**B** and **C**) Expression of C/G-specific CAR targets in primary-fusion-positive patients versus normal BM (NBM) (**B**) and C/G-CB versus GFP-CB cells (**C**). CBFA2T3-GLIS2 primary samples (*n* = 39); NBM samples (*n* = 68). For cultured cells, *n* = 4 technical replicates for C/G-CB cells in EC coculture at week; *n* = 3 technical replicates for all other groups. (**D**) Top: Gating strategies used to identify AML cells and normal lymphocytes, monocytes, and myeloid cells in 4 representative patients based on CD45 expression and SSC. Bottom: FOLR1 expression in the AML blast subpopulation versus normal cells. (**E**) Quantification of FOLR1 expression (geometric mean fluorescent intensity, MFI) among AML blasts and their normal counterparts across *n* = 15 patients. Autofluorescence was used as control. ****P < 0.00005 by 1-way ANOVA. (**F** and **G**) Expression of FOLR1 (**F**) and quantification of FOLR1^+^ cells (**G**) among GFP-CB and C/G-CB over weeks in EC coculture. Data presented as mean ± SD from 3 technical replicates.

Although chimeric antigen receptor (CAR) T therapy has proven successful in treating B cell acute lymphoblastic leukemia (B-ALL), immunotherapeutic targeting of AML remains a challenge given significant overlap of target antigens expressed on AML and normal hematopoietic cells. Our expansive target discovery effort through TARGET and Target Pediatric AML (TpAML) (8) has identified a library of AML-restricted genes (expression in AML, silent in normal hematopoiesis) in one or more AML subtypes, including C/G AML (607 genes; Figure 4A and Supplemental Figure 6). To determine which of these genes are expressed in C/G AML, we interrogated the transcriptome of C/G-CB cells cultured with ECs and primary C/G AML and determined upregulated genes that overlap between C/G-CB cells and primary C/G specimens. This analysis identified 42 candidates (among the 607 genes) that were significantly upregulated in both C/G-CB cells cultured with ECs and primary C/G AML, representing C/G-fusion-linked genes. Eighteen of these encode proteins that localize to the plasma membrane, of which 6 C/G-fusion-specific CAR targets (*FOLR1*, *MEGF10*, *HPSE2*, *KLRF2*, *PCDH19*, and *FRAS1*) were found to be highly expressed in C/G-CB cells and in C/G patients but entirely silent in normal hematopoiesis (Figure 4, B and C).

We prioritized folate receptor α (FOLR1) for further development given its existing record as a target in solid tumors (29). Further analysis showed that *FOLR1* is uniquely expressed in C/G AML (31 out of 39 [80%] of C/G-positive samples expressed *FOLR1* greater than 1 transcript per kilobase million [TPM]) and is silent in other AML and in normal bone marrow bulk samples and peripheral blood CD34^+^ samples (Supplemental Figure 7). We confirmed *FOLR1* transcript expression by qPCR (Supplemental Figure 8). Flow cytometric analysis of primary AML cells showed that FOLR1 was expressed on AML blasts but not on normal lymphocytes, monocytes, and myeloid cells within individual patients (Figure 4, D and E). We note that patient 4 had an expanded population of normal CD34^+^ cells (nonleukemic) that does not express FOLR1. Out of 15 C/G-positive samples, 12 (80%) exhibited FOLR1 cell surface expression. Cell surface FOLR1 protein was detected in C/G-CB cells as early as 6 weeks of EC coculture, progressing to near uniform expression by week 12 (Figure 4, F and G). Given that *FOLR2* (folate receptor β) is a paralog of *FOLR1*, we also assessed its expression in C/G AML. In contrast to *FOLR1*, *FOLR2* exhibited limited expression in C/G-positive patient samples and in the C/G-CB cells (Supplemental Figure 9, A and B). Flow cytometric analysis showed that cell surface FOLR2 was absent from the C/G-CB cells in EC coculture (Supplemental Figure 9C).

We next evaluated FOLR1 expression in normal tissues. FOLR1 staining was detected by immunohistochemistry in the renal tubular epithelium and luminal surface of pulmonary epithelium (patchy), raising concerns for on-target/off-tumor toxicity with FOLR1-targeted therapies (Supplemental Figure 10).

### Development and optimization of FOLR1-directed CAR T for C/G AML.

**Figure 5:**
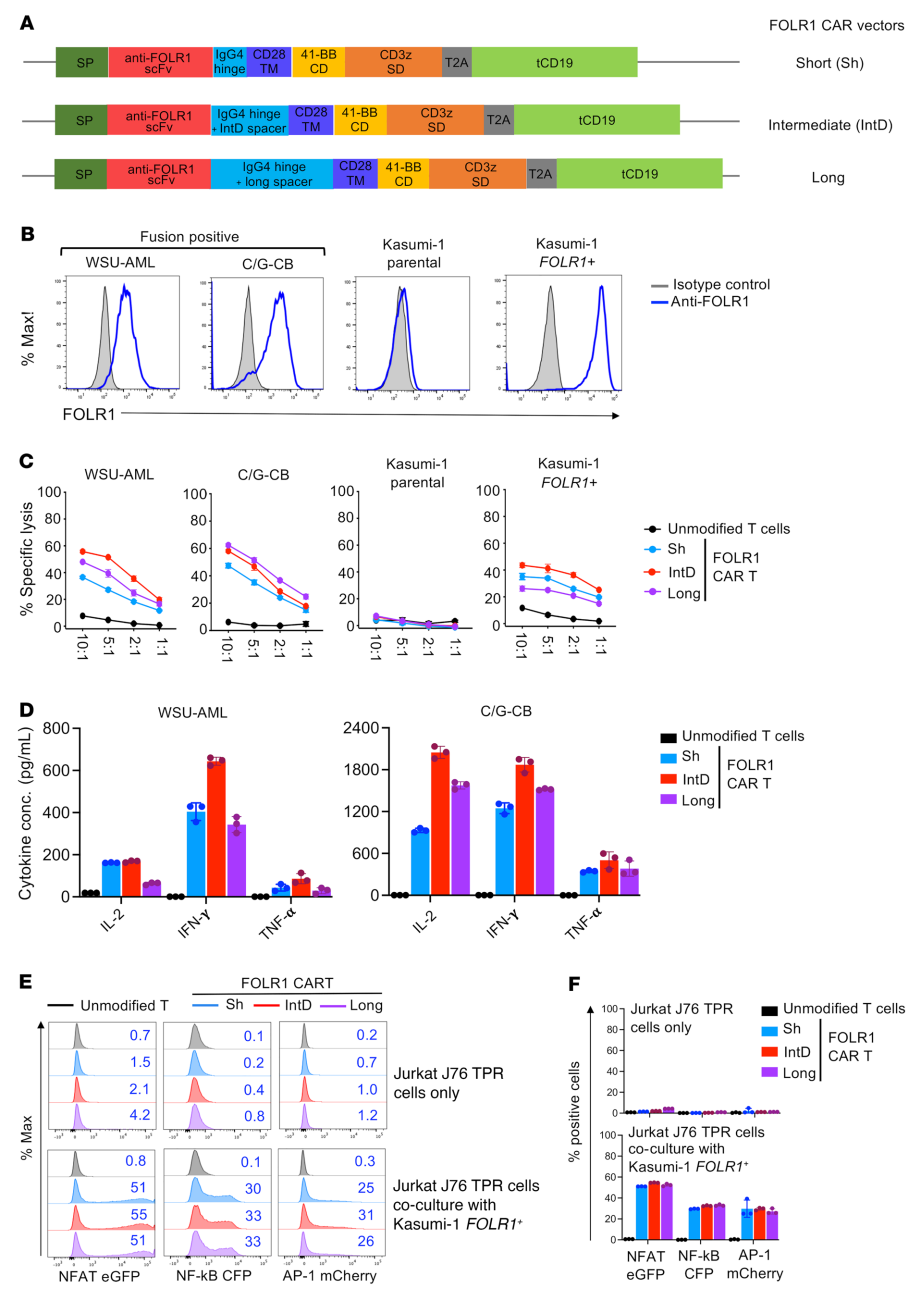
FOLR1 CAR constructs and reactivity of short, intermediate, and long FOLR1 CAR T cells. (**A**) Schematic diagram of second-generation FOLR1 CAR constructs with different IgG4 spacer lengths. SP, GM-CSFR signal peptide; scFv, single-chain variable fragment; TM, transmembrane domain; CD, costimulatory domain; SD, stimulatory domain; tCD19, transduced marker truncated CD19. (**B**) Expression of FOLR1 in C/G-CB, M07e, WSU-AML, Kasumi-1 *FOLR1^+^*, and Kasumi-1 parental cells. Blue = stained with PE-labeled anti-FOLR1; gray = isotype control. (**C**) Cytolytic activity of CD8^+^ T cells unmodified or transduced with short, intermediate, or long FOLR1 CAR construct against C/G-CB (cells taken >9 weeks in EC coculture), M07e, WSU-AML, Kasumi-1 *FOLR1*^+^, and Kasumi-1 parental cells in a 6-hour assay. Shown is the mean percentage specific lysis ± SD from 3 technical replicates at indicated effector/target (E:T) ratios. Data are representative of 3 donors. (**D**) Concentration of secreted IL-2, IFN-γ, and TNF-α in the supernatant following 24 hours of CD8^+^ T cell/AML coculture at 1:1 E:T ratio. Mean ± SD from 3 technical replicates is shown. Data are representative of 3 donors. (**E**) Representative flow plots showing expression of NFAT, NF-κB, and AP-1 in Jurkat J76 TPR reporter cells transduced with FOLR1 CAR constructs cultured alone (top) or coincubated with Kasumi-1 *FOLR1^+^* target cells for 24 hours at 1:1 E:T ratio (bottom). Kasumi-1 *FOLR1*^+^ cells were labeled with CellTrace Violet cell proliferation dye to differentiate from Jurkat cells. Transduced Jurkat cells were gated based on tCD19 expression. Number in top right corner indicates the percentage of positive cells. Analysis was performed on day 4 after transduction. (**F**) Quantification of percentage of NFAT^+^, NF-κB^+^, and AP-1^+^ cells in **E**. This experiment was repeated once.

The evidence that FOLR1 is causally linked to the C/G fusion and uniquely expressed in AML blasts suggested that targeting FOLR1 may provide a specific strategy to eliminate C/G leukemia without impacting normal hematopoiesis. To evaluate the therapeutic potential of targeting FOLR1, we generated FOLR1-directed CARs by fusing the single-chain variable fragment (scFv) derived from the anti-FOLR1 antibody farletuzumab to the IgG4 spacer with 3 different spacer lengths (short, intermediate, and long), CD28 transmembrane, 4-1BB costimulatory, and CD3ζ signaling domains (Figure 5A). We optimized the IgG4 spacer region against fusion-positive cell lines (WSU-AML and C/G-CB cells [>9 weeks in EC coculture]), Kasumi-1 cells engineered to express FOLR1 (Kasumi-1 *FOLR1^+^*), and Kasumi-1 parental cells (Figure 5B). Although all constructs conferred similar cytotoxicity against FOLR1^+^ cells, overall higher levels of proinflammatory cytokines (IL-2, IFN-γ, and TNF-α) were produced by CAR with intermediate relative to short and long IgG4 spacers (Figure 5, C and D). We assayed NFAT, NF-κB, and AP-1 expression in Jurkat J76 TPR reporter cells (30) transduced with the CAR constructs either cultured alone or cocultured with Kasumi-1 *FOLR1^+^* cells. None of the FOLR1 CAR constructs demonstrated tonic signaling in the absence of target binding (Figure 5, E and F).

### FOLR1 CAR T demonstrates preclinical efficacy in C/G AML.

**Figure 6:**
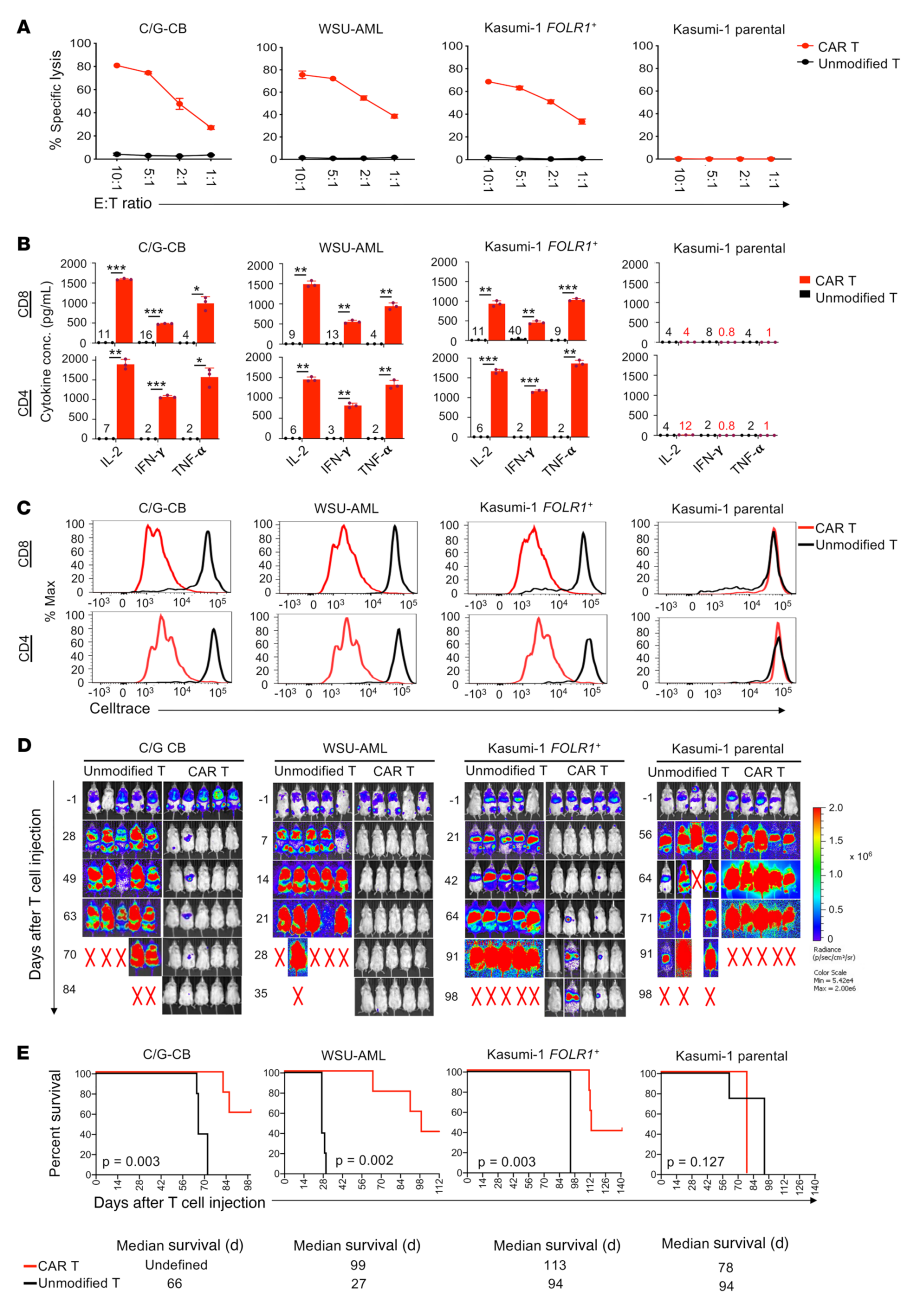
Preclinical efficacy of FOLR1 CAR T cells against C/G AML cells. (**A**) Cytolytic activity of CD8^+^ T cells unmodified or transduced with FOLR1 CAR following 6 hours of coculture with C/G-CB (cells taken after >9 weeks in EC coculture), WSU-AML, Kasumi-1 *FOLR1^+^*, and Kasumi-1 parental cells. Data presented are mean leukemia specific lysis ± SD from 3 technical replicates at indicated effector/target (E:T) ratios. Data are representative of 3 donors. (**B**) Concentration of secreted IL-2, IFN-γ, and TNF-α in the supernatant following 24 hours of T cell/AML coculture at 1:1 E:T ratio as measured by ELISA. Data are representative of 2 donors and are presented as mean ± SD from 3 technical replicates. Where concentrations of cytokines were too low to discern, the number above the *x* axis indicates the average concentration. Statistical significance was determined by unpaired, 2-tailed Student’s *t* test. *P < 0.05, **P < 0.005, ***P < 0.0005. Data are representative of 2 donors. (**C**) Representative flow cytometric analysis of cell proliferation of cell proliferation dye–labeled (CellTrace-labeled) unmodified and FOLR1 CAR T cells after 4-day coculture with target cells at a 1:1 E:T ratio. CAR T cells divided rapidly and diluted their CellTrace fluorescence after 4-hour coincubation with FOLR1^+^ AML cells. Data are representative of 2 donors. (**D**) Bioluminescence imaging of C/G-CB, WSU-AML, Kasumi-1 *FOLR1^+^*, and Kasumi-1 leukemias in mice treated with unmodified or FOLR1 CAR T cells at 5 × 10^6^ T cells per mouse. *n* = 5 mice/group. Radiance scale indicates an increase in leukemia from blue to red; X indicates death. (**E**) Kaplan-Meier survival curves of xenografts treated with unmodified or FOLR1 CAR T cells. *n* = 5 per group. Statistical differences in survival were evaluated using Mantel-Cox log-rank test. Note: 2 C/G-CB–bearing mice treated with CAR T cells died without leukemia and T cells present in bone marrow, spleen, and liver tissues and in peripheral blood as determined by flow cytometric analysis. *n* = 5 mice/group.

Using the FOLR1-directed CAR with the intermediate spacer, we further tested the target specificity of FOLR1-directed CAR T cells against FOLR1-positive (C/G-CB [taken after >9 weeks in EC coculture], WSU-AML, Kasumi-1 *FOLR1^+^*) and FOLR1-negative (Kasumi-1) cells. CD8^+^ FOLR1 CAR T cells demonstrated cytolytic activity against FOLR1-positive but not FOLR1-negative cells (Figure 6A), whereas unmodified and mesothelin (MSLN) CAR T cells, constructed using the same framework as FOLR1-directed CAR (as described in ref. 37), did not impact target cell viability (Figure 6A and Supplemental Figure 11A). Furthermore, both CD8^+^ and CD4^+^ FOLR1 CAR T cells produced higher levels of IL-2, IFN-γ, and TNF-α and proliferated more robustly than did unmodified or MSLN CAR T cells when coincubated with FOLR1-positive but not FOLR1-negative cells (Figure 6, B and C, and Supplemental Figure 11B). We confirmed that the MSLN CAR T cells were reactive to the Nomo-1 AML cell line, which expresses MLSN but not FOLR1 (Supplemental Figure 11C). Consistent with specificity, FOLR1 CAR T cells did not demonstrate reactivity against WSU-AML FOLR1-knockout cells (Supplemental Figure 11, D–F). These results indicate highly specific reactivity of FOLR1 CAR T cells against AML cells expressing FOLR1.

We next investigated the in vivo efficacy of FOLR1-directed CAR T cells. In C/G-CB (>9 weeks in EC coculture), WSU-AML, and Kasumi-1 *FOLR1^+^* xenograft models, treatment with FOLR1 CAR T cells resulted in leukemia clearance, while disease progression occurred in all mice that received unmodified T cells (Figure 6D and Supplemental Figure 12, A and B). Leukemia clearance was associated with expansion of CAR T cells in the peripheral blood of C/G-CB and WSU-AML xenografts (Supplemental Figure 12C). Importantly, treatment with FOLR1 CAR T cells significantly extended the median survival in mice bearing C/G-CB, WSU-AML, and Kasumi-1 *FOLR1^+^* leukemias (Figure 6E). The activity of FOLR1 CAR T cells in vivo was target specific, as they did not limit the leukemia progression nor extend the survival of Kasumi-1 xenografts (Figure 6, D and E). We note that in the WSU-AML–bearing mice that relapsed following CAR T treatment, the leukemia cells did express FOLR1 (Supplemental Figure 12, D and E).

### FOLR1 CAR T cells exhibit antileukemia activity against primary C/G-positive AML cells.

**Figure 7:**
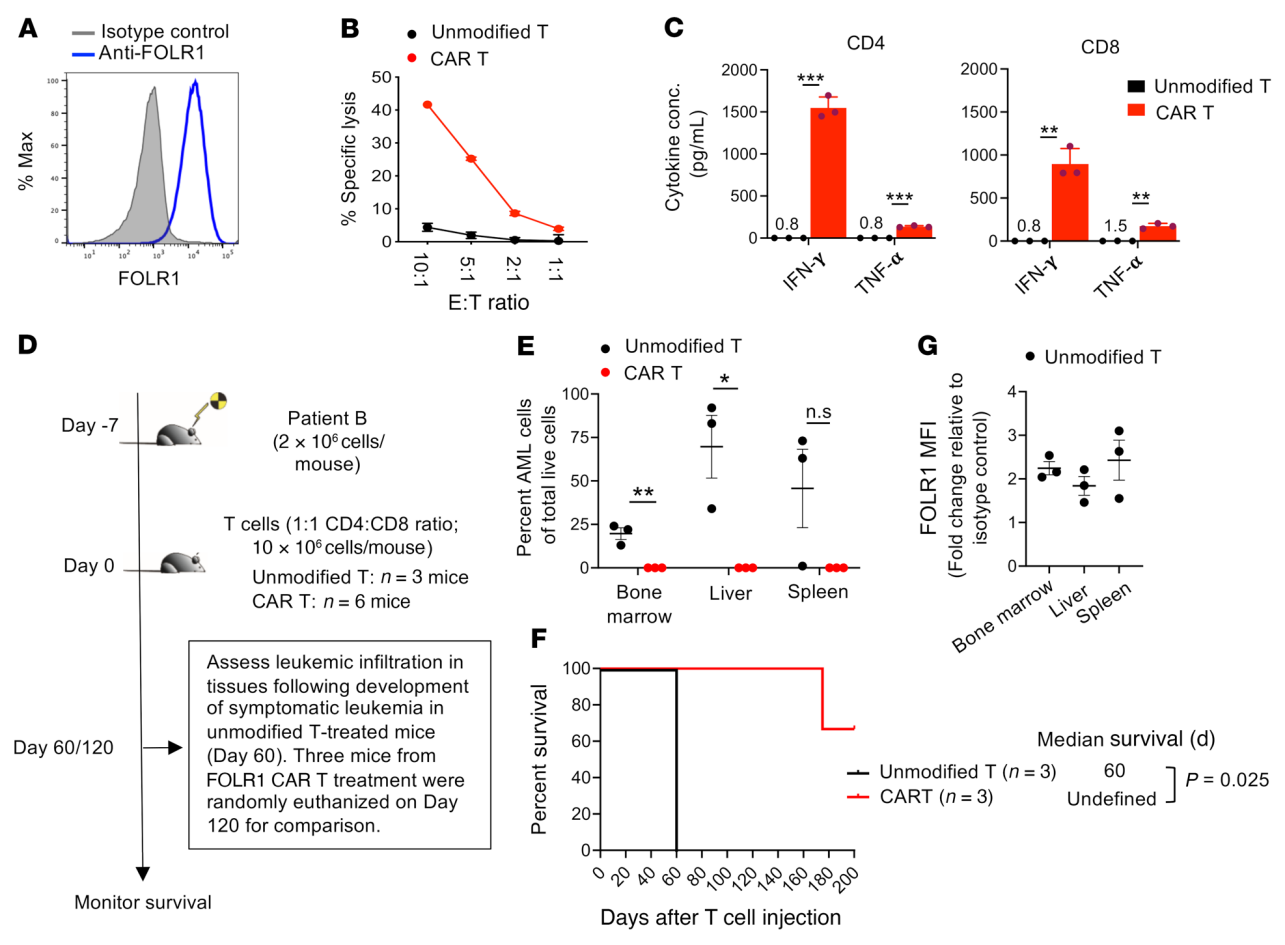
In vitro and in vivo assessment of FOLR1 CAR T cells against primary AML cells derived from a C/G-positive patient. (**A**) Expression of FOLR1 in primary AML cells from C/G-positive patient B (see Figure 1 for details on this patient). Blue = stained with PE-labeled anti-FOLR1; gray = isotype control. (**B**) Cytolytic activity of CD8^+^ T cells unmodified or transduced with FOLR1 CAR following 6 hours of coculture with patient B AML cells. Data presented are mean leukemia-specific lysis ± SD from 3 technical replicates at indicated effector/target (E:T) ratios. Shown is representative experiment out of 3 experiments (see Supplemental Figure 14 for results from 2 additional donors). (**C**) Concentration of secreted IFN-γ and TNF-α in the supernatant following 24 hours of T cell/AML coculture at a 1:1 E:T ratio as measured by ELISA. Data are presented as mean ± SD from 3 technical replicates. Where concentrations of cytokines were too low to discern, the number above the *x* axis indicates the average concentration. Statistical significance was determined by unpaired, 2-tailed Student’s *t* test. **P < 0.005, ***P < 0.0005. Shown is representative experiment out of 2 experiments. (**D**) Schematic of experiment evaluating in vivo efficacy of FOLR1 CAR T cells against primary AML cells from patient B. Only 1 experiment was performed due to limited sample. (**E**) Bone marrow, liver, and spleen were harvested from control mice at necropsy following development of leukemia (60 days after T cell injection) as well as from 3 FOLR1 CAR T cell–treated mice selected at random 120 days after T cell injection. Percentage AML cells (defined as CD45^dim^CD56^+^) in the bone marrow, liver, and spleen are shown. *n* = 3 mice per group. Error bars denote ± SEM. Statistical significance was determined by unpaired, 2-tailed Student’s *t* test, assuming unequal variances. *P < 0.05, **P < 0.005. (**F**) Expression of FOLR1 among AML cells in the bone marrow, liver, and spleen from mice treated with unmodified T cells at necropsy. *n* = 3 mice per group. Error bars denote ± SEM. (**G**) Kaplan-Meier survival curves of PDX mice treated with unmodified or FOLR1 CAR T cells. Statistical differences in survival were evaluated using Mantel-Cox log-rank test. Only 1 experiment was performed for in vivo assessment. *n* = 3 mice per group.

To evaluate the antileukemia activity of FOLR1 CAR T cells in primary C/G-positive AML, we utilized bone marrow cells isolated from patient B, which exhibited uniform and relatively high expression of FOLR1 (Figure 7A and Supplemental Figure 13). Coincubation of FOLR1 CAR T cells with patient bone marrow cells resulted in cytolysis of AML cells (Figure 7B and Supplemental Figure 14) and increased secretion of IFN-γ and TNF-α compared with unmodified T cells (Figure 7C), demonstrating sensitivity of the primary AML cells to FOLR1 CAR T cell–mediated killing. In vivo activity was next assessed by transplanting 2 × 10^6^ of patient B’s bone marrow cells per mouse into NSG-SGM3 mice. After 1 week, unmodified or FOLR1 CAR T cells were injected into the leukemia-bearing mice at 1 × 10^7^ cells (1:1 CD4^+^/CD8^+^ cell ratio) per mouse (Figure 7D). Mice treated with unmodified T cells developed symptomatic leukemia with significant engraftment of AML in the bone marrow, liver, and spleen at 60 days following T cell injection, whereas AML engraftment was undetectable in the CAR T–treated mice on day 120 after T cell injection (Figure 7E). Importantly, treatment with FOLR1 CAR T cells led to a significant increase in survival of the PDX mice (*P* = 0.025; Figure 7F). We confirmed FOLR1 expression by the AML cells that engrafted in the unmodified T cell–treated mice and in one of the CAR T–treated mice that developed symptomatic leukemia on day 180 after T cell injection (Figure 7G and Supplemental Figure 15). Together, these results show that the FOLR1 CAR T cells were effective at eliminating primary C/G-positive AML cells in vitro and in vivo.

### FOLR1 CAR T cells do not affect viability, self-renewal, and multilineage differentiation of normal HPSCs.

**Figure 8:**
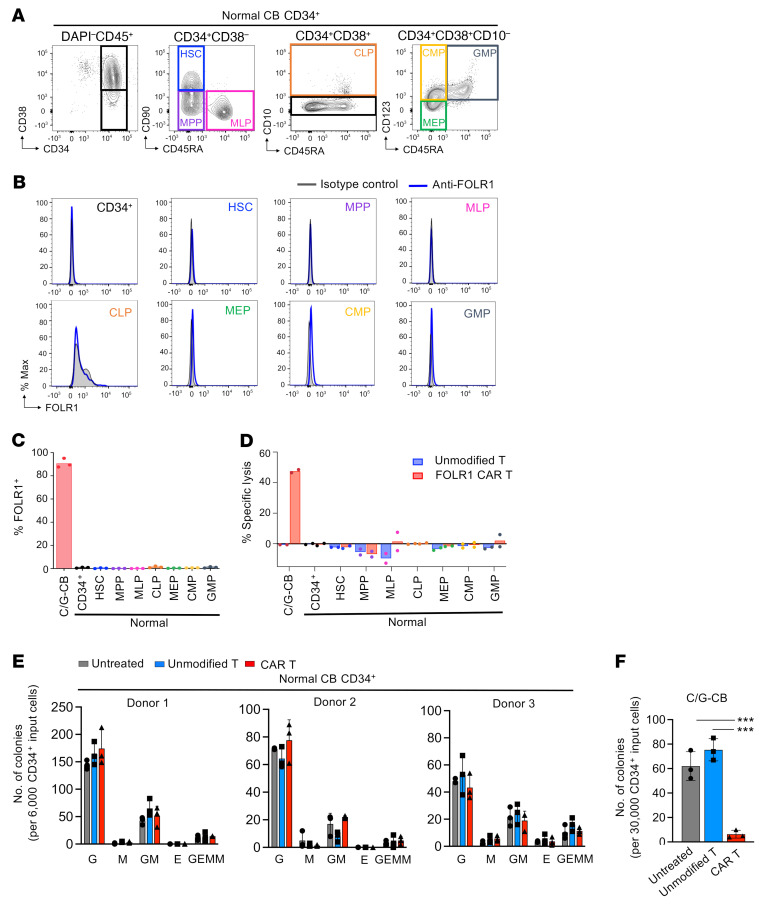
FOLR1-directed CAR T cells effectively eliminate C/G-CB cells without affecting viability of HSPCs. (**A**) Gating strategy used to identify HPSC subsets from a representative CD34-enriched bone marrow sample from a healthy donor. Shown is representative of 3 donors. Immunophenotype of the HSPCs is as follows: CD34^+^CD38^–^CD90^+^CD45RA^–^ (hematopoietic stem cells, HSCs); CD34^+^CD38^–^CD90^–^CD45RA^–^ (multipotent progenitors, MPPs); CD34^+^CD38^–^CD90^–^CD45RA^+^ multilymphoid progenitors, MLPs); CD34^+^CD38^+^CD10^+^ (common lymphoid progenitors, CLPs); CD34^+^CD38^+^CD10^–^CD123^–^CD45RA^–^ (megakaryocyte-erythroid progenitors, MEPs); CD34^+^CD38^+^CD10^–^CD123^+^CD45RA^–^ (common myeloid progenitors, CMPs); CD34^+^CD38^+^CD10^–^CD123^+^CD45RA^+^ (granulocyte monocyte progenitors, GMPs). (**B**) Histogram of FOLR1 expression in normal HSPC subsets. (**C**) Quantification of percentage FOLR1^+^ among C/G-CB cells (>12 weeks of EC coculture) and HSPC subsets from 3 CD34-enriched samples from healthy donors. (**D**) Percentage specific lysis in C/G-CB cells and the HSPC subsets shown in **C** following 4-hour incubation with unmodified or FOLR1 CAR T cells at a 2:1 E:T ratio. Note that data points for C/G-CB cells are from 2 technical replicates. Only 2 out of 3 normal CD34^+^ samples were used in this experiment. (**E** and **F**) After 4 hours, cocultures of healthy donor CD34^+^ or C/G-CB cells with either unmodified or FOLR1 CAR T cells at a 2:1 E:T ratio were transferred to methylcellulose with cytokines for colony-forming cell (CFC) assay. (**E**) Colonies derived from erythroid (E), granulocyte-macrophage (G, M, and GM), and multipotential granulocyte, erythroid, macrophage, megakaryocyte (GEMM) progenitors were scored and enumerated after 7 to 10 days. (**F**) Total colonies from C/G-CB cells are tabulated. Data are presented as mean ± SD from 3 technical replicates for each donor. No significant difference was detected between cocultures with unmodified T cells versus FOLR1 CAR T cells for normal HSPCs for each colony type. Statistical significance was determined by 1-way ANOVA. ***P < 0.0005.

To determine whether FOLR1 is expressed on normal HSPCs, we characterized FOLR1 expression in CD34^+^ CB samples from 3 healthy donors. FOLR1 expression was entirely silent in HSPC subsets (Figure 8, A–C). Consistent with lack of expression, no cytolytic activity was detected against HPSCs after 4-hour coincubation with FOLR1 CAR T cells (Figure 8D). Moreover, FOLR1 CAR T cells did not affect the self-renewal and multilineage differentiation capacity of normal HSPCs as compared to unmodified control T cells (Figure 8E), whereas significant eradication of colonies was detected in the C/G-CB cells (Figure 8E). Taken together, these results suggest that FOLR1 CAR T cells can eradicate C/G AML cells without compromising normal HSPCs and may be a promising therapy for C/G AML.

## Discussion

In this study, we demonstrate that the C/G oncogenic fusion promotes transformation of human CB HSPCs that recapitulates the transcriptome, morphology, and immunophenotype of infant C/G AMKL and a xenograph model of C/G AML. We further demonstrate that a coculture system with ECs can promote leukemic transformation and preserves self-renewal for over 90 days. The ECs provide the necessary microenvironmental support that synergizes with the fusion to produce a megakaryoblastic leukemia that closely resembles primary C/G AMKL. Together, these results show that driver oncogenic fusions in pediatric AML may be sufficient to induce the AML phenotype in an appropriate developmental context (CB HSPCs) and a permissive microenvironment. Importantly, we used this model to identify potential therapeutic targets. We have identified FOLR1 as a unique and specific target expressed in C/G AMKL and developed a potent CAR T cell that effectively eliminates C/G leukemia cells while sparing normal HPSCs, providing a potential therapeutic approach for this high-risk AML subtype.

Given the prenatal origin and the lack of recurrent cooperating mutations associated with the C/G fusion, our results support the hypothesis that certain driver oncogenic fusions are sufficient to promote leukemic transformation in pediatric AML, which contrasts with the widely accepted cooperativity model of AML in adults, requiring synergy of a class II (fusion) and class I (single-nucleotide variants, SNVs) variants for (transformation to AML) (31). Furthermore, our evidence that transduced CB HPSCs generated a leukemia that is almost entirely AMKL corroborates a previous study, reported by Lopez et al., showing that early developmental hematopoiesis favors megakaryoblastic transformation (14). That same study also demonstrated that the differentiation stage of the transformed cells influences lineage phenotype, where transformation at the HSC or lineage-bias multipotential progenitor (MPP) stage can affect whether the fusion will confer the AMKL versus AML phenotype. In normal hematopoiesis, HSCs occupy the apex of the hematopoietic hierarchy giving rise to all blood cells, while MPP2, MPP3, and MPP4 cells predominantly give rise to erythroid-megakaryocytic cells, granulocyte-monocyte myeloid cells, and lymphoid cells, respectively (32). Using an inducible C/G mouse model, Lopez et al. (15) showed that transformation of fetal liver HSCs and MPP2 cells produces CD41^+^ AMKL, whereas transformation of fetal liver MPP3 cells gives rise to both CD41^+^ and granulocyte marker 1^+^ (Gr1^+^) leukemia cells. This contrasts with adult bone marrow, where transformation of HSCs results in CD41^+^ leukemia, while transformation of MPP2, MPP3, and MMP4 cells results in both CD41^+^ and Gr1^+^ leukemias, with Gr1^+^ cells being more predominate in the transformed MPP3 and MPP4 populations. We plan to further investigate the mechanisms by which ontogeny plays a role in directing the subtypes of human C/G leukemia in future studies with C/G-transformed fetal liver, CB, and adult HSPCs cultured in the EC coculture system.

Most patients with C/G-fusion AML are either refractory to therapy or maintain minimal residual disease following treatment (9, 10, 33). Clinical features of C/G AML include extensive bone marrow involvement at diagnosis (33, 34), suggesting that the interaction with the bone marrow microenvironment may contribute to resistance to standard chemotherapies. Consistent with this, we previously identified a number of cell adhesion molecules that exhibited elevated expression in C/G patient samples compared with normal bone marrow counterparts and other AML subtypes (8), further implicating cell-cell interactions in C/G AML biology and resistance. In addition, our studies show C/G AMKL is a focal disease with tight aggregates of malignant cells with a cohesive appearance that are more similar to a solid tumor than most hematologic neoplasms. In less cellular areas there was a unique pattern of focal adhesion with intercellular bridges between the malignant C/G-CB cells in the bone marrow of transplanted mice and the PDX model. A similar histologic pattern is also present in PDX models reported by Thioller et al. (7). Transcriptional profiling of C/G-CB cells identified upregulation of several cell adhesion molecules and integrins, suggesting that cell-cell interactions may be critical for establishing the tight tumor clusters observed in vivo. Furthermore, C/G-CB cells in culture require continuous direct interactions with ECs for sustained proliferation, underscoring the importance of AML/EC interactions in the leukemic process. These interactions among C/G leukemia cells and between the C/G leukemia cells and the EC niche may also promote drug resistance. Further studies of the precise cell-cell interactions that are critical for leukemic transformation and drug resistance, facilitated by the EC coculture system presented here, will provide new mechanistic insights into the disease biology of C/G AML, and help define novel therapeutic strategies to eradicate this aggressive leukemia.

The results presented here also address a fundamental challenge in immunotherapy for AML, as AML-restricted targets have been elusive. By integrating transcriptomics of C/G-CB cells and primary C/G AML, we have identified 6 C/G-fusion-specific genes that represent potential high-value targets and validated the specific cell surface expression of FOLR1. Therapeutic targeting of FOLR1 using various approaches (small molecule, folate-drug conjugate, antibody-drug conjugate, and CAR T cells) is currently under investigation and is the focus of several clinical trials for solid tumors (see ref. 29 and ClinicalTrials.gov), providing initial efficacy and safety data. Here, we have shown that FOLR1 represents a promising target in C/G AML, as RNA-seq analysis of primary specimens showed unique expression in C/G AML without significant expression in normal HSPCs and bulk bone marrow samples, providing an ideal target for immunotherapy. Flow cytometric analysis of primary patient specimens confirmed that FOLR1 surface expression was restricted to AML blasts and absent on normal lymphoid, monocytic, myeloid, and CD34^+^ cells in the patients’ bone marrow. C/G-CB cells also demonstrated robust and uniform expression of FOLR1 on the cell surface following long-term EC coculture. Consistent with target expression, FOLR1-directed CAR T cells effectively eradicated C/G AML cells, including cells derived from a primary patient specimen, but did not affect the viability, self-renewal, and multilineage differentiation of normal HSPCs. Since FOLR1-directed CAR T cells do not impact normal hematopoiesis, targeting FOLR1 would in principle not lead to prolonged myelosuppression or myeloablation, which are significant barriers for current CAR T therapy in AML targeting lineage markers CD33 and CD123. Together, these results provide the preclinical foundation for further evaluation of FOLR-directed CAR T as a potential therapeutic approach for C/G AML. Future evaluation must address potential on-target, off-tumor toxicity of FOLR1 CAR T cells, as FOLR1 is expressed in healthy tissues (kidney, intestine, lung, retina, placenta, and choroid plexus; ref. 35), and prior studies with a T cell bispecific antibody targeting FOLR1 have demonstrated severe lung toxicity in nonhuman primates (36). Consistent with previous reports, we also detected FOLR1 expression in the renal tubular epithelium and the pulmonary epithelial cells. A possible strategy to circumvent on-target off-tumor toxicity is the use of dual targeting/AND-gated CARs requiring binding to both FOLR1 and a hematopoietic cell–specific marker to elicit T cell–mediated killing of tumor cells.

In this study, we demonstrate potent, target-specific cytotoxicity of FOLR1 CAR T cells in vitro against a variety of AML cell lines characterized by various levels of FOLR1 expression. Further optimization of CAR T functionality will be necessary, because despite initial clearance of the leukemia in the WSU-AML and Kasumi-1 *FOLR1*^+^ xenograft mice to below the level of detection by IVIS imaging in response to treatment with FOLR1 CAR T cells (5 × 10^6^ cells/mouse), the treated mice eventually relapsed. We have previously shown that increasing the cellular dose of CAR T cells can have a significant impact on the in vivo efficacy (37), and perhaps increasing the number of CAR T cells may improve the in vivo antileukemia activity against FOLR1^+^ AML. On the other hand, antigen density may limit CAR T functionality. Additional work is required to determine whether antigen density affects CAR T cell activity and the susceptibility of the leukemia cells to CAR T cell cytotoxicity. If antigen density proves to affect CAR functionality, modifying CAR elements such as scFv binding and signaling components (i.e., CD28ζ) can enhance sensitivity and cytotoxicity (38) and should be evaluated in C/G AML.

Progress in elucidating mechanisms of disease and development of novel therapies for the C/G AML cohort is currently limited by a lack of relevant human model systems that accurately recapitulate human disease. The EC coculture platform we developed overcomes this barrier and recapitulates the vascular EC niche that supports malignant transformation, self-renewal, and propagation of leukemia-generating cells in vitro. This platform is thus suited to interrogating AML-niche interactions and identifying novel therapeutic targets for C/G, and it should be extended to studies with other oncogenic fusions.

## Methods

### Animals.

NOD/SCID/γ*c^−/−^* (NSG) and NOD.Cg-*Prkdc^scid^ Il2rg^tm1Wjl^* Tg (CMV-IL3,CSF2,KITLG)1Eav/MloySzJ (NSG-SGM3) mice were obtained from The Jackson Laboratory and housed and bred at the Fred Hutchinson Cancer Research Center (FHCRC). For all experiments, 6- to 10-week-old age-matched females were randomly assigned to experimental groups. Mice transplanted with C/G-CB or AML cell lines were monitored and euthanized when they exhibited symptomatic leukemia (tachypnea, hunchback, persistent weight loss, fatigue, or hind-limb paralysis).

### Primary specimens.

Human umbilical CB samples were obtained from normal deliveries at Swedish Medical Center (Seattle, Washington, USA). Frozen aliquots of AML diagnostic bone marrow samples were obtained from the Children’s Oncology Group. Cells were thawed in Iscove’s modified Dulbecco’s medium (IMDM) supplemented with 20% FBS and 100 U/mL DNase I (Sigma-Aldrich, D5025). A bone marrow biopsy was obtained from a C/G patient treated at the University of Minnesota Masonic Children’s Hospital. Healthy donor T cells were obtained from Bloodworks Northwest. We confirmed that these cells lacked infectious agents (EBV, HCMV, hepatitis A, hepatitis B, hepatitis C, HHV 6, HHV 8, HIV1, HIV2, HPV16, HPV18, HSV1, HSV2, HTLV 1, HTLV 2, and *Mycoplasma* spp.) through IDEXX Bioanalytics.

### Cell lines.

M07e (DSMZ, ACC104), WSU-AML (BioIVT, HCL-WSUAML-AC), and Kasumi-1 (ATCC, CRL-2724) cell lines were maintained per vendors’ instructions. All parental cell lines used for these studies were purchased from commercial vendors and upon initial expansion, multiple vials were frozen for future use. Cell lines were used at early passage. Short tandem repeat and chromosomal analysis testing at the Specimen Processing Shared Resource at FHCRC were used to validate these cell lines. Cells are also routinely tested for mycoplasma contamination using the MycoAlert Mycoplasma Detection Kit from Lonza Corporation. We generated WSU-AML FOLR1-knockout cells using CRISPR gene editing by following the manufacturer’s protocol. The sgRNA targeting exon 1 was designed using the Broad Institute’s GPP sgRNA Design tool (https://portals.broadinstitute.org/gpp/public/analysis-tools/sgrna-design) and synthesized by Synthego with the sequence 5′-CAUGAACGCCAAGCACCACA-3′ plus modified scaffold. Transfection of WSU-AML cells was performed using Lonza Nucleofector Kit C (VCA-1004) with Cas9 protein from Invitrogen (A36498). We engineered the Kasumi-1 *FOLR1*^+^ cell line by transducing Kasumi-1 cells with a lentivirus containing the *FOLR1* transgene driven by the *EF1a* promoter (Genecopoeia, LPP-C0250-Lv156-050). Jurkat J76 TPR reporter cells (30) were maintained in RPMI supplemented with 20% FBS and 2 mM L-glutamine.

### Transduction of CD34^+^ CB cells.

CB samples were processed with red blood cell lysis buffer and enriched for CD34^+^ cells using CliniMACS CD34 MicroBeads (Miltenyi Biotec, 130-017-501). CD34^+^ CB cells were then seeded onto plates coated with RetroNectin (5 μg/mL; Takara, T100A) plus Notch ligand Delta1 (2.5 μg/mL; ref. 39) overnight in SFEM II medium (StemCell Technologies, 09650FH) containing 50 ng/mL stem cell factor (SCF; StemCell Technologies, 78062), 50 ng/mL thrombopoietin (TPO; StemCell Technologies, 78210), and 50 ng/mL Fms-like tyrosine kinase 3 ligand (FLT3L; StemCell Technologies, 78009). Cells were transduced the following day with the C/G construct at an MOI of 200 or GFP control construct at MOI of 50. Transduced cells were grown on Notch ligand at 37°C in 5% CO_2_ for 6 days and then sorted for GFP^+^ cells. Sorted GFP^+^ cells were either transplanted into NSG-SGM3 mice at 200,000 cells per mouse or placed in EC coculture or MC (see ref. 23 and below) for long-term culture at 75,000 cells per well in a 6-well plate. In subsequent experiments using a CD34^+^ CB sample from another donor (CB 2, see Supplemental Figure 4), transduced cells were grown on Notch ligand for 2 days prior to placement in EC coculture or MC plating at 100,000 cells per well of a 12-well plate.

### Long-term culture of transduced CD34^+^ CB cells.

Transduced cells were placed in either EC coculture with SFEM II medium supplemented with 50 ng/mL SCF, 50 ng/mL TPO, 50 ng/mL FLT3L, and 100 U/mL penicillin-streptomycin (Pen/Strep), or MC-containing IMDM (Gibco, 12-440-053) supplemented with 15% FBS (Corning, 35-010-CV), 100 U/mL Pen/Strep (Gibco, 15-140-122), 10 ng/mL SCF, 10 ng/mL TPO, 10 ng/mL FLT3L, 10 ng/mL IL-6 (Shenandoah Biotechnology, 100-10), and 10 ng/mL IL-3 (Shenandoah Biotechnology, 100-80). For EC cocultures, HUVECs transduced with the E4ORF1 construct (E4 ECs) were propagated as previously described (17, 40). One day prior to coculture, E4 ECs were seeded into 6-well or 12-well plates at 800,000 or 300,000 cells per well, respectively, and cultured in medium 199 (Biowhittaker, 12-117Q) supplemented with 20% FBS (Hyclone, SH30088.03), endothelial mitogen (Biomedical Technologies, BT203), heparin (Sigma-Aldrich, H3149), HEPES (Gibco, 15630080), L-glutamine (Gibco, 25030), and Pen/Strep. After 24 hours, E4 ECs were washed with PBS and cultured with transduced CB cells in media described above. Transduced CB cells in either culture condition were propagated with fresh media and E4 ECs replaced every week until cells stopped proliferating. Three to twenty percent of the cultures were replated each week for long-term culture.

We confirmed *C/G* and *FOLR1* expression in C/G-CB cells over weeks in culture using RT-PCR (Supplemental Figures 8 and 16). Transduced CB cells were sorted for GFP^+^ cells on a FACSAria II using FACSDiva Software (BD Biosciences). DNA and RNA from sorted cells were extracted with AllPrep DNA/RNA/miRNA Universal Kit using the QIAcube platform (QIAGEN). Expression of the fusion transcript in GFP^+^ cells was confirmed by RT-qPCR TaqMan assay and QuantStudio 5 real-time PCR system (Thermo Fisher Scientific) using the primers Forward 5′-CCCTGACGGTCATCAACCA-3′, Reverse 5′-CACCATCCAAATAGCGCAGTG-3′, and TaqMan probe 5′-[FAM]-CAGCGAGGACTTCCAG-[MGB]-3′. *FOLR1* expression was determined using an RT-qPCR TaqMan assay (Thermo Fisher Scientific, Hs01124177_m1, 4331182).

### RNA-seq analysis.

Total RNA was extracted using the QIAcube automated system with AllPrep DNA/RNA/miRNA Universal Kits (QIAGEN, 80224) for diagnostic pediatric AML samples from peripheral blood or bone marrow, as well as bulk healthy bone marrows and healthy CD34^+^ peripheral blood samples. Total RNA from C/G-CB and GFP-CB cells in EC coculture and MC at indicated time points was purified as described above. The 75-bp strand-specific paired-end mRNA libraries were prepared using the ribodepletion 2.0 protocol by the British Columbia Genome Sciences Center (BCGSC, Vancouver, British Columbia, Canada) and sequenced on the Illumina HiSeq 2000/2500. Sequenced reads were quantified using Kallisto v0.45.0 (41) with a GRCh38 transcriptome reference prepared using the coding and noncoding transcript annotations in Gencode v29 and RepBase v24.01, and gene-level counts and abundances were produced using tximport v1.16.1 (42). See supplemental material for details of transcriptome analysis.

### Generation of human FOLR1 CAR T cells.

CAR T cells were generated by transducing healthy donor T cells (Bloodworks Northwest) with lentivirus carrying the FOLR1 CAR vectors. Peripheral blood mononuclear cells from healthy donors were isolated over Lymphoprep (StemCell Technologies, 07851). CD4^+^ or CD8^+^ T cells were isolated by negative magnetic selection using EasySep Human CD4^+^ T cell Isolation Kit II (StemCell Technologies, 17952) and EasySep Human CD8^+^ T cell Isolation Kit II (StemCell Technologies, 17953). Purified T cells were cultured in CTL media (RPMI supplemented with 10% human serum [Bloodworks Northwest], 2% L-glutamine [Gibco, 25030-081], 1% Pen/Strep [Gibco, 15140-122], 50 nM β-mercaptoethanol [Gibco, 21985-023], and 50 U/mL IL-2 [aldesleukin, Prometheus]) at 37°C in 5% CO_2_. T cells were activated with anti-CD3/anti-CD28 beads (3:1 beads/cell; Gibco, 11131D) on RetroNectin-coated plates (5 μg/mL, coated overnight at 4°C; Takara, T100B) and transduced with CAR lentivirus (MOI = 50) 1 day after activation via spinoculation at 800*g* for 90 minutes at 25°C in CTL media (with 50 U/mL IL-2) supplemented with 8 μg/mL protamine sulfate. Transduction used 200,000 cells per well in 24-well plates. Transduced cells were expanded in CTL media (with 50 U/mL IL-2) and separated from beads on day 5. As truncated CD19 was coexpressed with the CAR by a T2A ribosomal skip element, it was used to select for transduced cells. Transduction efficiency was approximately 30% to 40% for CD8^+^ cells and 55% to 80% for CD4^+^ cells (see representative data in Supplemental Figure 17). Transduced cells were sorted for CD19 expression (using anti–human CD19 microbeads [Miltenyi Biotec, 130-050-301]) on an Automacs 8–10 days after activation. Sorted cells were further expanded in CTL (with 50 U/mL IL-2) media for an additional 4 to 6 days prior to in vitro and in vivo cytotoxicity assays.

### In vitro cytotoxicity studies.

Target cells (C/G-CB >9 weeks in EC coculture, M07e, WSU-AML, Kasumi-1 *FOLR1*^+^, and Kasumi-1 parental) were split 1 to 2 days prior to cytotoxicity assay. Target leukemia cells were labeled with 2.5 μM CFSE (Invitrogen, C34554) per manufacturer’s protocol, washed with 1× PBS, and resuspended in CTL media (without IL-2). For T cell proliferation assay, effector cells (unmodified or CAR T cells) were labeled with 2.5 μM CellTrace Violet cell proliferation Dye (Invitrogen, C34557), washed with 1× PBS, serially diluted in CTL media (without IL-2), and combined with target cells at various effector/target (E:T) ratios in 96-well U-bottom plates. Cytotoxicity (at indicated time points) and T cell proliferation (4 days) were assessed by flow cytometry after staining cells with live/dead fixable viability dyes (FVDs; Invitrogen, L34964 [cytotoxicity] or L10120 [T cell proliferation]). Percentage dead among target cells was assessed by gating on FVD^+^ among CFSE^+^ target cells. Percentage specific lysis was calculated by subtracting the average of the 3 replicate wells containing target cells only from each well containing target and effector cells at each E:T ratio. After 24 hours of coculture, media supernatant was assessed for IL-2, IFN-γ, and TNF-α production by Luminex microbead technology (provided by FHCRC Immune Monitoring Core).

### In vivo cytotoxicity studies.

Target leukemia cells were transduced with mCherry/luciferase (C/G-CB, weeks 9–12 in EC coculture; plasmid 104833, Addgene) or eGFP/luciferase construct (WSU-AML, Kasumi-1 *FOLR1*^+^, and Kasumi-1 parental; plasmid 104834, Addgene) and sorted for mCherry^+^ or GFP^+^ cells, respectively. Luciferase-expressing cells were injected intravenously into NSG-SGM3 (C/G-CB) at 5 × 10^6^ cells per mouse or NSG (WSU-AML, Kasumi-1 *FOLR1*^+^, and Kasumi-1 parental) mice at 1 × 10^6^ cells through the tail vein. Mice were treated with FOLR1 CAR T or unmodified T cells via tail vein intravenous injection 1 week following leukemia cell injection. Leukemia burden was measured by bioluminescence imaging weekly. Leukemia burden and T cell expansion were monitored by flow cytometric analysis of mouse peripheral blood, which was drawn by retro-orbital bleeds for the indicated time points starting from the first week of T cell injection. Flow cytometric analysis of peripheral blood and tissues was performed as described above (see supplemental material for antibodies).

### Data and code availability.

RNA-seq data on primary patient samples are deposited at the National Cancer Institute’s Genomic Data Commons (https://gdc.cancer.gov/) under the TARGET-AML project. Additionally, sequence files can be accessed from the Database of Genotypes and Phenotypes (https://www.ncbi.nlm.nih.gov/gap/) under accession number phs000465.v19.p8. RNA-seq data on engineered CB are deposited in NCBI’s Gene Expression Omnibus (GEO GSE181726). All codes used in this study are publicly available.

### Statistics.

An unpaired, 2-tailed Student’s *t* test was used to determine statistical significance for all in vitro studies. For comparisons between more than 2 groups, 1-way ANOVA was performed where indicated. Log-rank (Mantel-Cox) test was used to compare Kaplan-Meier survival curves between experimental groups. Statistical significance was defined as *P* less than 0.05.

Box-and-whisker plots denote the median (middle line) with 25th (lower bound of box) and 75th percentiles (upper bound of box) as hinges; upper and lower whiskers extend from the hinge to 1.5 × IQR (interquartile range). All biological replicates’ expression data were plotted, and outliers are those with expression values beyond the whiskers. 

### Study approval.

Experiments were performed after approval by Institutional Animal Care and Use Committee of the FHCRC (protocol 51068) and in accordance with institutional and national guidelines and regulations. All human specimens used in this study were obtained after written informed consent from patients and donors. The research was performed after approval by the FHCRC Institutional Review Board (protocol 9950). The study was conducted in accordance with the Declaration of Helsinki.

## Author contributions

QL designed the experiments. QL, TAH, JS, AL, BJH, AML, SC, TTT, CNM, L Perkins, L Pardo, JLS, AKB, RW, RI, SF, TI, LC, FP, SMM, JMO, and RR performed the experiments and analyzed the data. SS, SI, MRL, LB, SRR, IDB, FM, and KT provided general scientific guidance and performed data analysis. QL, BH, KRL, and SM wrote the manuscript. All authors reviewed the manuscript prior to submission.

## Supplementary Material

Supplemental data

## Version 1 09/22/2022

In-Press Preview

## Version 2 11/15/2022

Electronic publication

## Version 3 08/15/2024

Corrected Supplemental Figure 12 per corrigendum
