# CBFA2T3-GLIS2 model of pediatric acute megakaryoblastic leukemia identifies FOLR1 as a CAR T cell target

**DOI:** 10.1172/JCI184305

**Published:** 2024-08-15

**Authors:** Quy Le, Brandon Hadland, Jenny L. Smith, Amanda Leonti, Benjamin J. Huang, Rhonda Ries, Tiffany A. Hylkema, Sommer Castro, Thao T. Tang, Cyd N. McKay, LaKeisha Perkins, Laura Pardo, Jay Sarthy, Amy K. Beckman, Robin Williams, Rhonda Idemmili, Scott Furlan, Takashi Ishida, Lindsey Call, Shivani Srivastava, Anisha M. Loeb, Filippo Milano, Suzan Imren, Shelli M. Morris, Fiona Pakiam, Jim M. Olson, Michael R. Loken, Lisa Brodersen, Stanley R. Riddell, Katherine Tarlock, Irwin D. Bernstein, Keith R. Loeb, Soheil Meshinchi

Original citation: *J Clin Invest*. 2022;132(22):e157101. https://doi.org/10.1172/JCI157101

Citation for this corrigendum: *J Clin Invest*. 2024;134(16):e184305. https://doi.org/10.1172/JCI184305

During the preparation of Supplemental Figure 12A, an image for day 35 was inadvertently used instead of an image for day 28. The supplemental file has been corrected.

The authors regret the error. 

## Version 1 08/15/2024

Electronic publication

