# Supplementary material for: CBFA2T3-GLIS2 model of pediatric acute megakaryoblastic leukemia identifies FOLR1 as a CAR T cell target
Source: J Clin Invest. 2022 Nov 15;132(22):e157101. doi: 10.1172/JCI157101 (PMC9663156; doi:10.1172/JCI157101)
Supplement: Supplemental data [file jci-132-157101-s009.pdf]

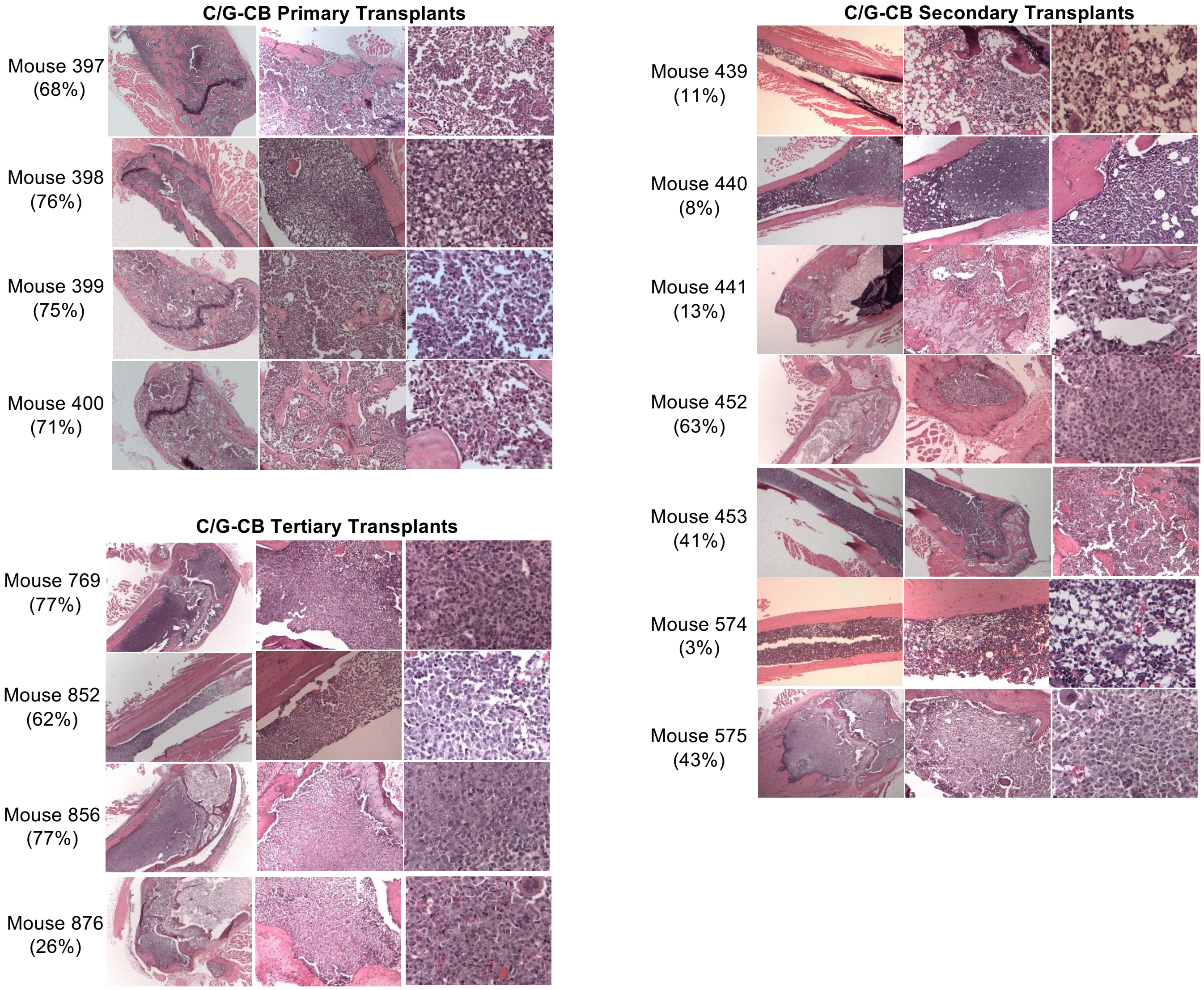

Supplemental Figure 1. C/G-CB cells form tight clusters in mouse bone marrow. (Related to Figure 1) H&E stains of femurs taken from primary, secondary and tertiary transplants of C/G-CB cells. Magnification: 2.5X(left), 10X (middle), 40X (right).

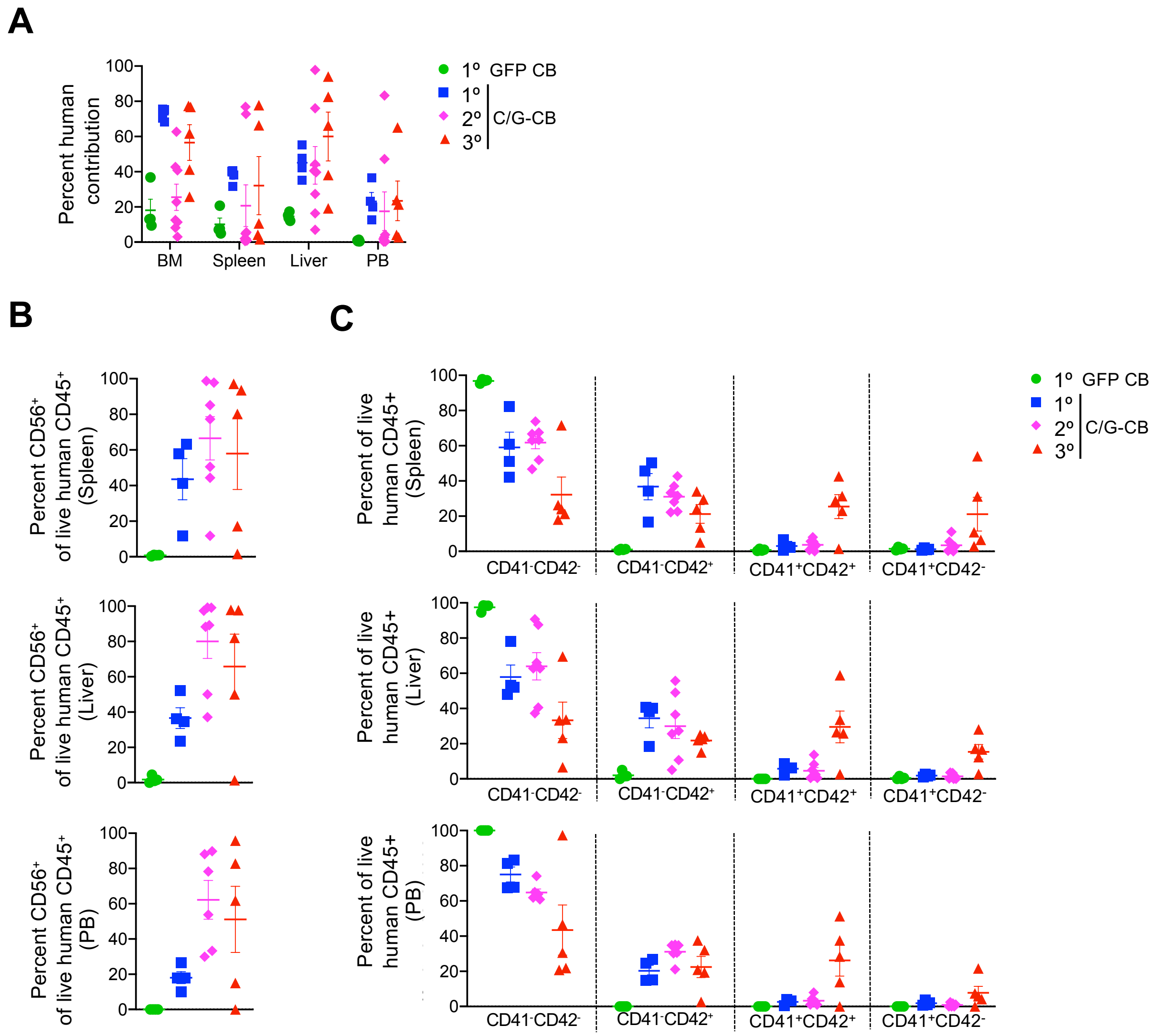

Supplemental Figure 2. Expression of CD56 and AMKL markers in C/G-CB xenograft cells following development of symptomatic leukemia in NSG-SGM3 mice. (Related to Figure 1) A. Percent human $CD45^+$ cells in the bone marrow, spleen, liver and peripheral blood (PB) from mice transplanted with C/G-CB cells in primary (1°, N=4 mice), secondary (2°, N=7 mice) and tertiary (3°, N=5 mice) transplants. **B, C.** Percent $CD56^+$ and CD41/CD42 subsets in mouse tissues described in A. 1°, N=4 mice per group; 2°, N=7 mice; and 3°, N=5 mice.

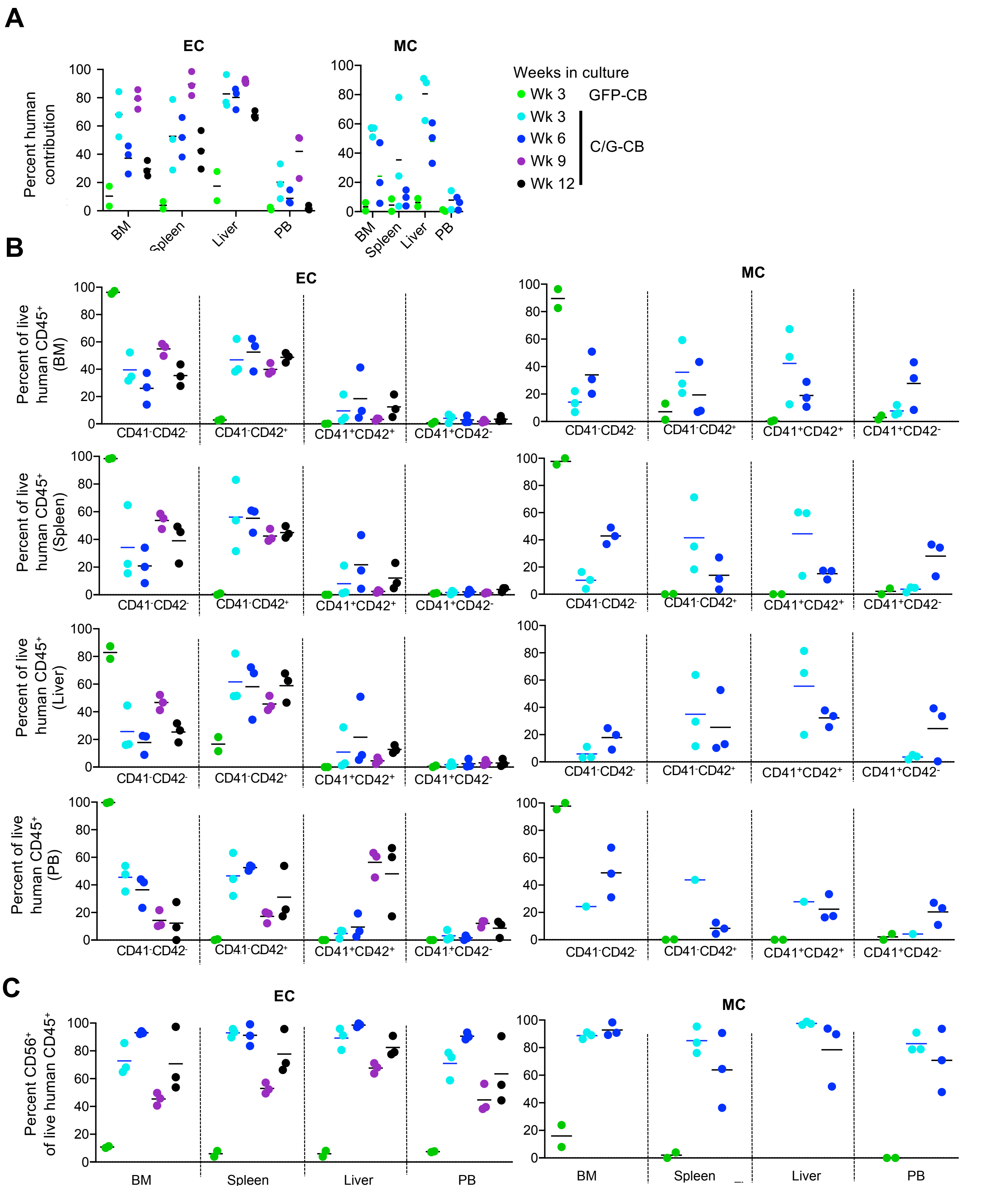

Supplemental Figure 3. Assessment of RAM and AMKL markers in C/G-CB cells isolated from mice transplanted with engineered cells cultured in EC co-culture or MC. (Related to Figure 2) A. Percent human CD45+ cells in the bone marrow, spleen liver and peripheral blood from mice transplanted with C/G-CB and GFP-CB cells at indicated timepoints in EC co-culture or MC. **B, C.** Percent CD41/CD42 subsets (B) andCD56+ cells (C) among live human CD45+ in mouse tissues described in A. Data analyzing CB cells in the liver for mice transplanted with GFP-CB cells from MC culture are not included as not enough cells were presence in these samples. Same goes for peripheral blood data from 2 mice transplanted with C/G-CB cells grown in MC. C/G-CB (N=3 mice/group), GFP-CB (N=2 mice/group)

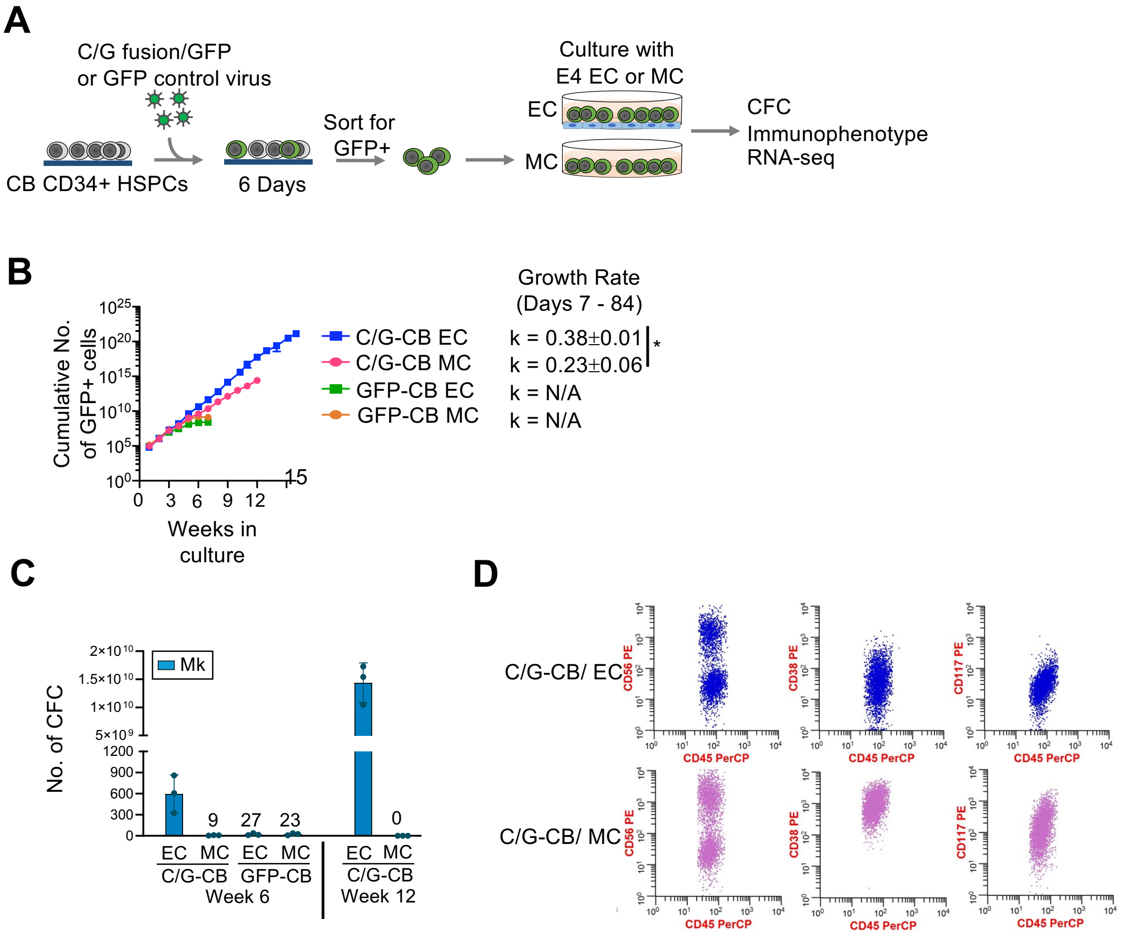

Supplemental Figure 4. ECs promote transformation of C/G-CB cells. (Related to Figure 2) A. Schematic of transduction and long-term cultures of cord blood CD34+ HSPCs from a second donor. **B.** Growth kinetics of transduced cells over days in EC or MC as determined by the cumulative number of GFP+ cells. Mean +/- standard deviation from 3 technical replicates are shown. Growth rate constant k was determined by regression analysis using the formula $N(t) = N(0)e^{kt}$ where t is measured in days. **C.** Following 6 and 12 weeks of culture, a fraction of each culture was transferred to megacult to enumerate Mk colonies. Data are normalized to the CD34+ input cells at the start of the culture and presented as mean +/- standard deviation from 3 technical replicates. **D.** Expression of the RAM immunophenotype in C/G-CB cells after 6 weeks in either EC co-culture or MC.

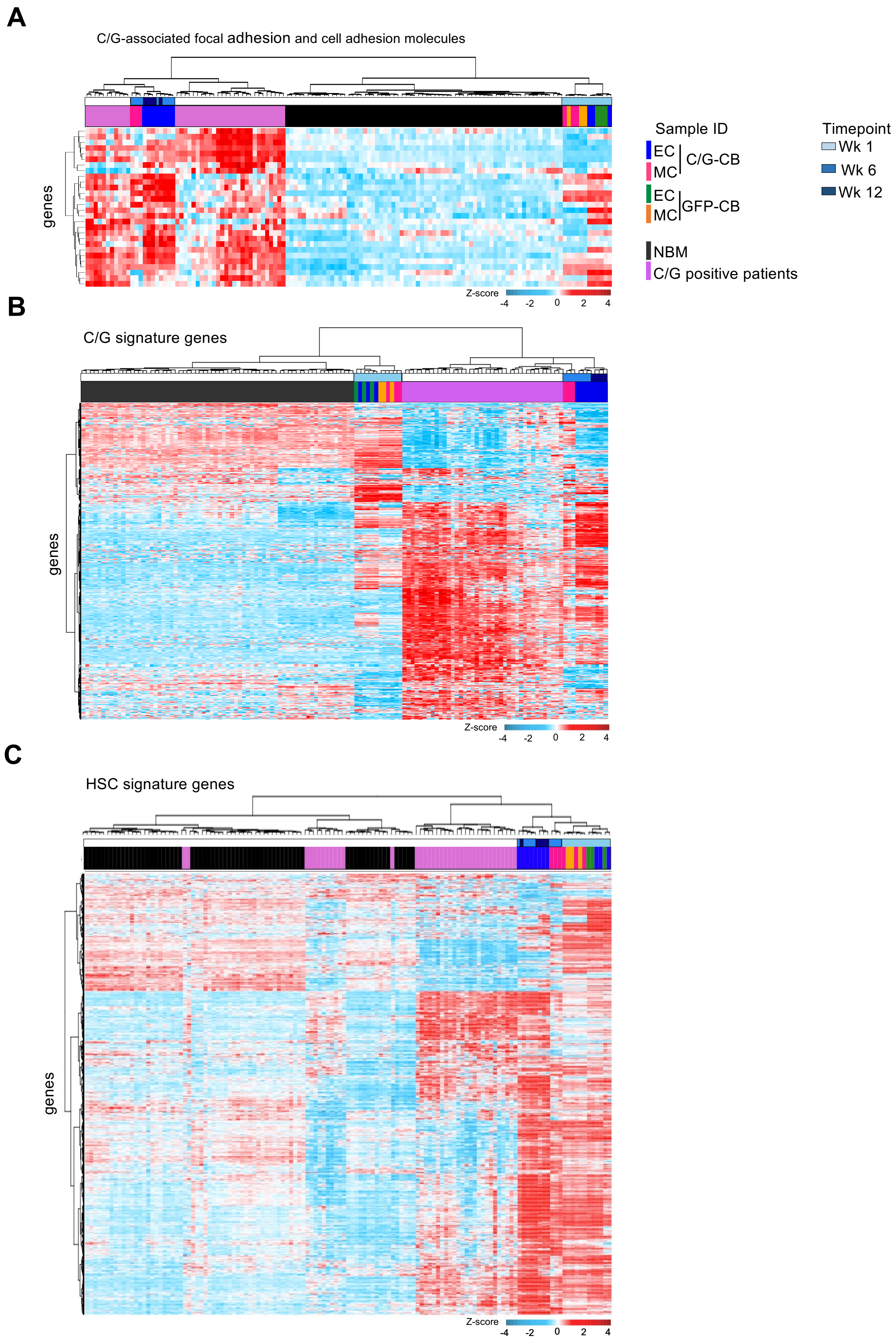

Supplemental Figure 5

Supplemental Figure 5. Expression of C/G-specific genes. (Related to Figure 3) Heat maps showing expression of C/G-specific focal adhesion and cell adhesion molecule genes (**A**), genes associated with primary C/G fusion positive AML (**B**), and HSC signature genes (**C**). Unsupervised hierarchical clustering demonstrates clustering of C/G-CB cells cultured with ECs for 6 and 12 weeks with primary C/G samples. CBFA2T3-GLIS2 primary samples (N=39); NBM samples (N=68). For cultured cells, N=4 technical replicates for C/G-CB cells in EC co-culture at week; N=3 technical replicates for all other groups.

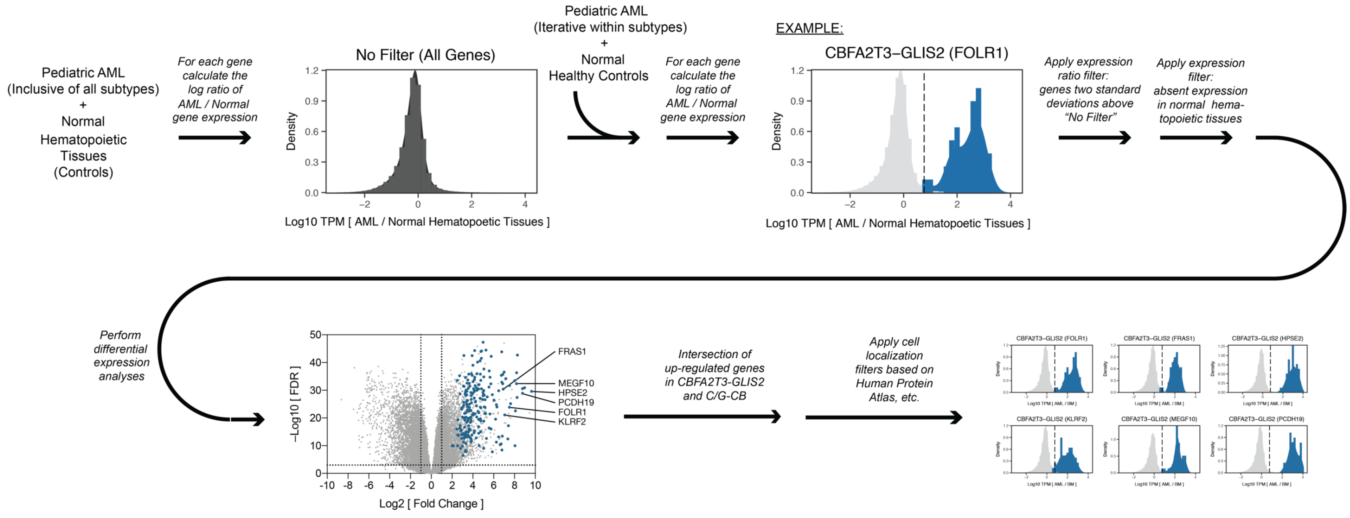

Supplemental Figure 6. Identification of C/G fusion-specific CAR targets. (Related to Figure 4A) Flow diagram of AML-restricted gene and CAR-T target identification. The procedure involves three main steps: 1) Determine the ratio of expression for AML primary samples versus healthy normal hematopoietic tissue samples (bulk marrows and CD34+ peripheral blood) from log10 transformed normalized gene expression. The ratio is calculated per gene from mean AML expression and mean normal hematopoietic tissue expression, where normal tissue values are the divisor, which acts as a measure of over or under expression. A normal curve is fit to the ratios and this procedure is completed for all heterogenous AML samples as a group, and iteratively within fusion and mutation subtypes; genes with ratios greater than +2 standard deviations and with absent expression in normal hematopoietic tissues were retained (N=607) for further analysis. 2) The AML restricted genes were further selected if found to be significantly overexpressed in fusion positive patient samples compared to healthy marrows and were likewise overexpressed in C/G-CB at weeks 6 and 12 in EC co-culture with absent expression in GFP-CB controls, providing several candidate (N=42) targets. 3) Optimal CAR-T targets were selected by the identification of candidate genes with cell surface localization potential, and those with an absence of expression in healthy tissue controls as noted in step 1, but expression in > 75% of C/G patient samples, and with moderate to high expression levels (N=6).

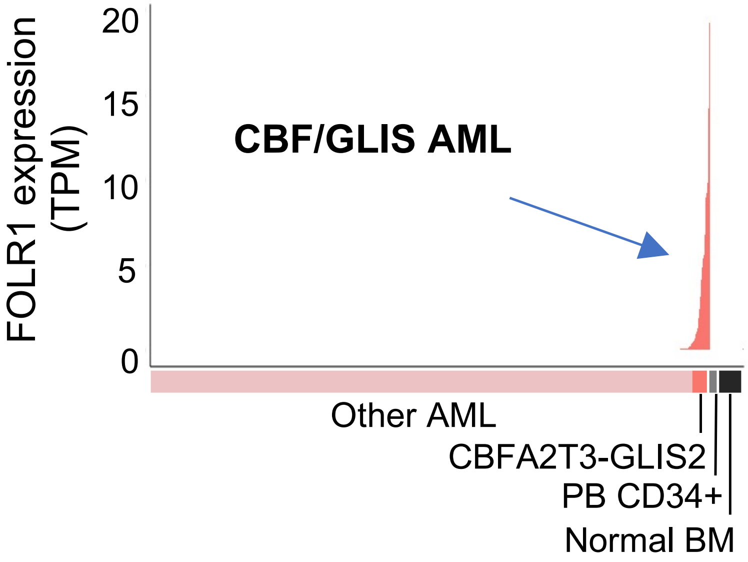

Supplemental Figure 7. *FOLR1* transcript is uniquely expressed in C/G AML. Waterplot showing transcript expression (TPM) among C/G AML (red, N=39), other AML (pink, N=1,453), peripheral blood (PB) CD34+ samples (grey, N=16) and bulk bone marrow samples (black, N=68).

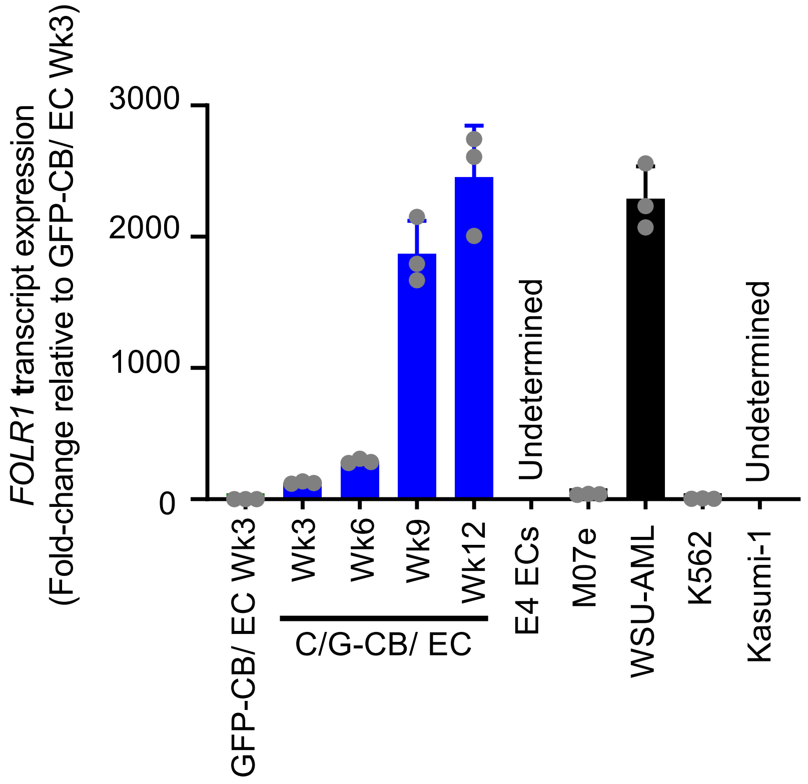

Supplemental Figure 8. Expression of *FOLR1* transcript in C/G-CB cells cultured on ECs. RT-PCR analysis of *FOLR1* expression in engineered CB cells and in fusion positive cell lines M07e and WSU-AML. Expression is normalized as fold-change relative to GFP-CB/EC Wk 3 samples. Data shown is the average of 3 technical replicates. Error bars denote standard deviation.

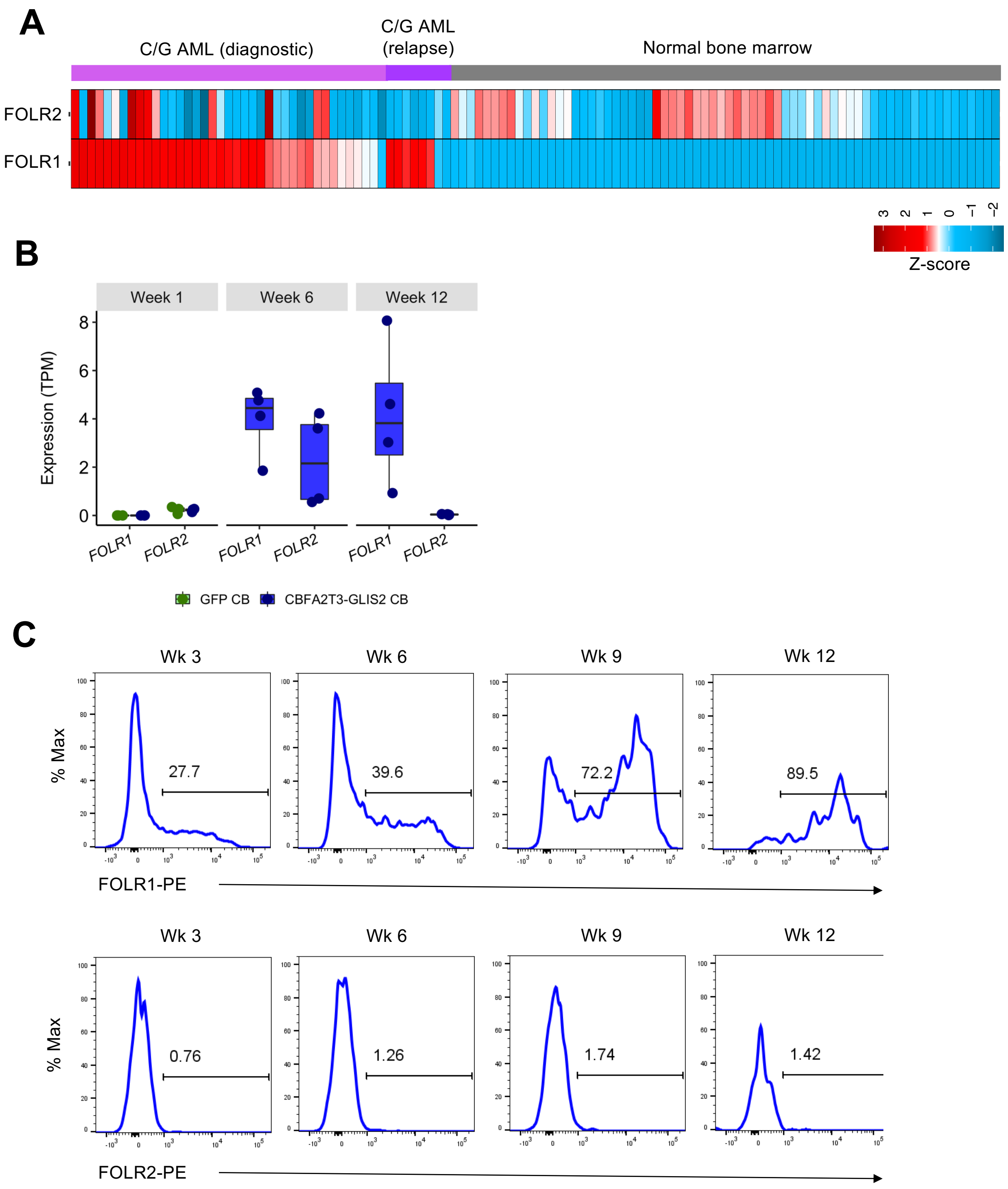

Supplemental Figure 9. Expression of FOLR2 (folate receptor beta) in C/G AML. A. Heat map showing expression of FOLR1 and FOLR2 among C/G-positive patients at diagnostic and relapse, and in bone marrow samples from healthy donors. **B, C.** Transcript (B) and cell surface (C) expression of FORL1 and FOLR2 in C/G-CB and GFP-CB cells over weeks in EC co-culture.

	1	2	3	4	5	6	7	8	9	10
A	Cerebrum	Cerebrum	Cerebrum	Cerebrum	Cerebrum	Cerebrum	Cerebellum	Cerebellum	Cerebellum	
B	Adrenal gland	Adrenal gland	Adrenal gland	Ovary	Ovary	Ovary	Pancreas	Pancreas	Pancreas	
C	Lymph node	Lymph node	Lymph node	Hypophysis	Hypophysis	Hypophysis	Testis	Testis	Testis	
D	Thyroid gland	Thyroid gland	Thyroid gland	Breast	Breast	Breast	Spleen	Spleen	Spleen	
E	Tonsil	Tonsil	Tonsil	Thymus gland	Thymus gland	Thymus gland	Bone marrow	Bone marrow	Bone marrow	
F	Lung	Lung	Lung	Heart	Heart	Heart	Esophagus	Esophagus	Esophagus	
G	Stomach	Stomach	Stomach	Small intestine	Small intestine	Small intestine	Colon	Colon	Colon	
H	Liver	Liver	Liver	Salivary gland	Salivary gland	Salivary gland	Kidney	Kidney	Kidney	
I	Prostate	Prostate	Prostate	Uterus	Uterus	Uterus	Cervix	Cervix	Cervix	
J	Striated muscle	Striated muscle	Striated muscle	Skin	Skin	Skin	Nerve	Nerve	Nerve	
K	Diaphragm	Diaphragm	Pericardium	Eye	Eye	Eye	Larynx	Larynx	Larynx	Adrenal gland (malig)

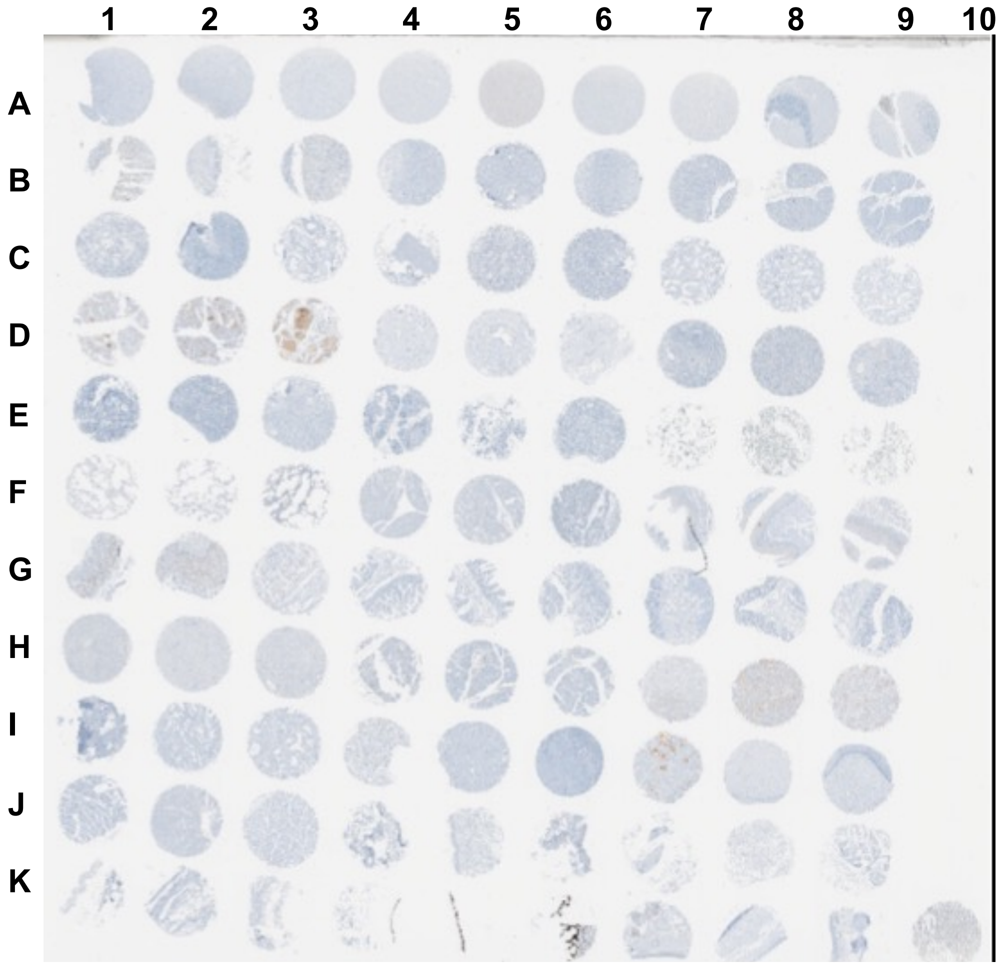

Supplemental Figure 10. Expression of FOLR1 in normal tissues. Immunohistochemistry of FOLR1 staining in 32 types of normal human organs (Tissue Microarray, TMA FDA999u), each type taken from 3 different individuals. Top, sample ID; bottom, FOLR1-stained TMA.

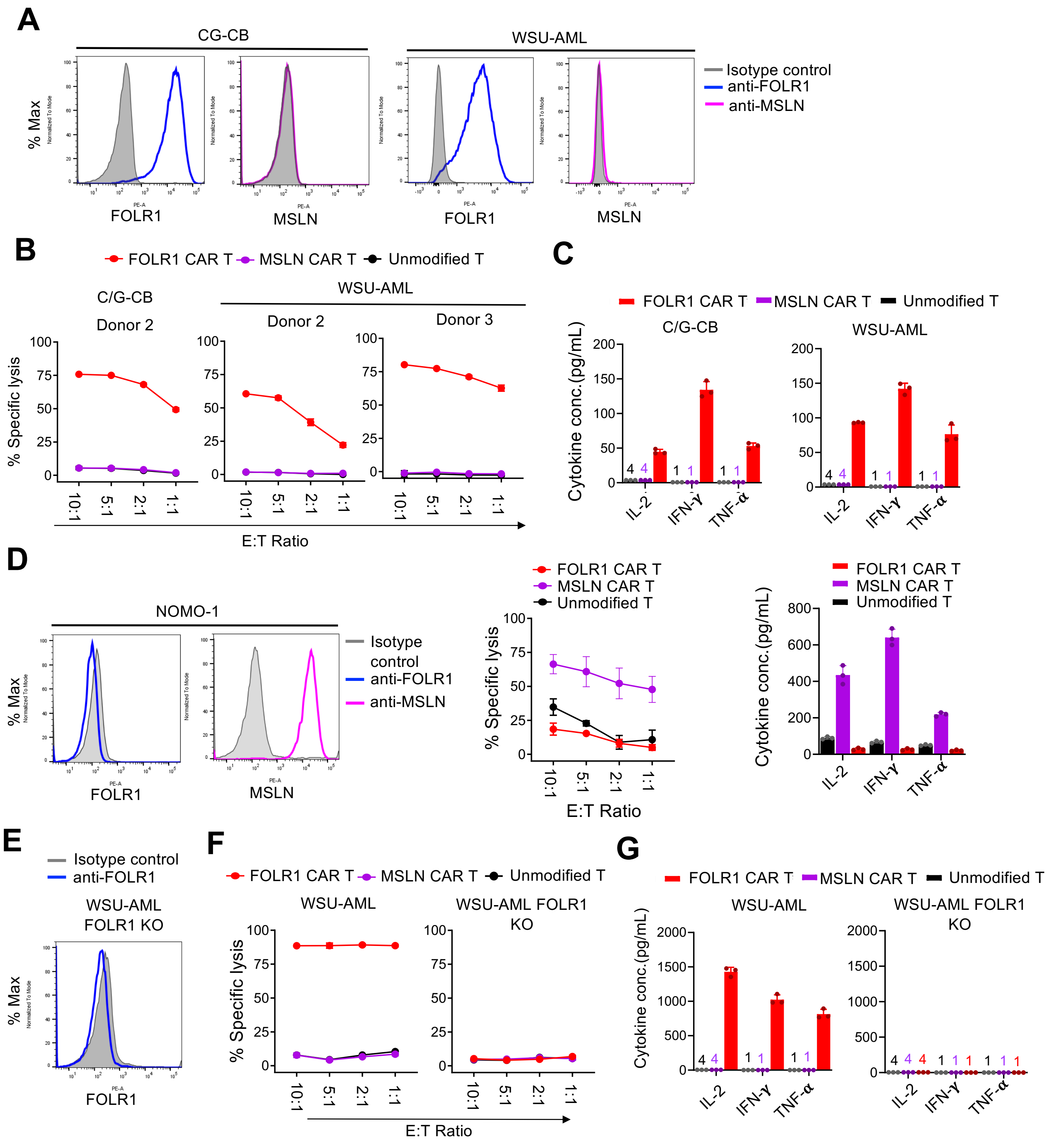
A
CG-CB
WSU-AML
% Max
FOLR1
MSLN
FOLR1
MSLN
Isotype control
anti-FOLR1
anti-MSLN
B
FOLR1 CAR T
MSLN CAR T
Unmodified T
C/G-CB
WSU-AML
Donor 2
Donor 2
Donor 3
% Specific lysis
E:T Ratio
C
FOLR1 CAR T
MSLN CAR T
Unmodified T
C/G-CB
WSU-AML
Cytokine conc.(pg/mL)
IL-2
IFN-γ
TNF-α
D
NOMO-1
% Max
FOLR1
MSLN
Isotype control
anti-FOLR1
anti-MSLN
FOLR1 CAR T
MSLN CAR T
Unmodified T
% Specific lysis
E:T Ratio
FOLR1 CAR T
MSLN CAR T
Unmodified T
Cytokine conc.(pg/mL)
IL-2
IFN-γ
TNF-α
E
Isotype control
anti-FOLR1
WSU-AML FOLR1 KO
% Max
FOLR1
F
FOLR1 CAR T
MSLN CAR T
Unmodified T
WSU-AML
WSU-AML FOLR1 KO
% Specific lysis
E:T Ratio
G
FOLR1 CAR T
MSLN CAR T
Unmodified T
WSU-AML
WSU-AML FOLR1 KO
Cytokine conc.(pg/mL)
IL-2
IFN-γ
TNF-α

Supplemental Figure 11. In vitro reactivity of FOLR1 CAR T cells against C/G AML cells. A. Flow cytometric analysis of FOLR1 and MSLN expression in C/G-CB cells and WSU-AML cell line. **B.** Cytolytic activity of CD8 T cells unmodified or transduced with FOLR1 CAR or MSLN CAR following 6 hours of co-culture with C/G-CB (cell taken from >9 weeks in EC co-culture) or WSU-AML. Data presented are mean leukemia specific lysis +/- SD from 3 technical replicates at indicated effector: target (E:T) ratios. T cells were derived from two additional donors. **C.** Concentration of secreted IL-2, IFN-γ, and TNF-α in the supernatant following 24 hour of T cell/AML co-culture at 1:1 E:T ratio as measured by ELISA. Data are derived from T cells isolated from donor 2 and are presented as mean +/- SD from 3 technical replicates. Where concentrations of cytokines are too low to discern, the number above the x-axis indicates the average concentration. **D.** Left, flow cytometric analysis of FOLR1 and MSLN expression in Nomo-1 AML cell line. Middle, cytolytic activity of CD8 T cells unmodified or transduced with FOLR1 CAR or MSLN CAR following 24 hours of co-culture with Nomo-1 cells. Data presented are mean leukemia specific lysis +/- SD from 3 technical replicates at indicated E:T ratios. Right, concentration of secreted IL-2, IFN-γ, and TNF-α in the supernatant following 24 hour of T cell/AML co-culture at 1:1 E:T ratio as measured by ELISA. Data are derived from T cells isolated from donor 2. **E.** Flow cytometric analysis of FORL1 in WSU-AML FOLR1 knock out (KO). **F.** Cytolytic activity of CD8 T cells unmodified or transduced with FOLR1 CAR or MSLN CAR following 24 hours of co-culture with WSU-AML cells or WSU-AML FOLR1 KO cells. Data are derived from T cells isolated from donor 2 and are presented as mean +/- SD from 3 technical replicates. **G.** Concentration of secreted IL-2, IFN-γ, and TNF-α in the supernatant following 24 hour of T cell/AML co-culture at 1:1 E:T ratio as measured by ELISA. Data are derived from T cells isolated from donor 2 and are presented as mean +/- SD from 3 technical replicates. Where concentrations of cytokines are too low to discern, the number above the x-axis indicates the average concentration.

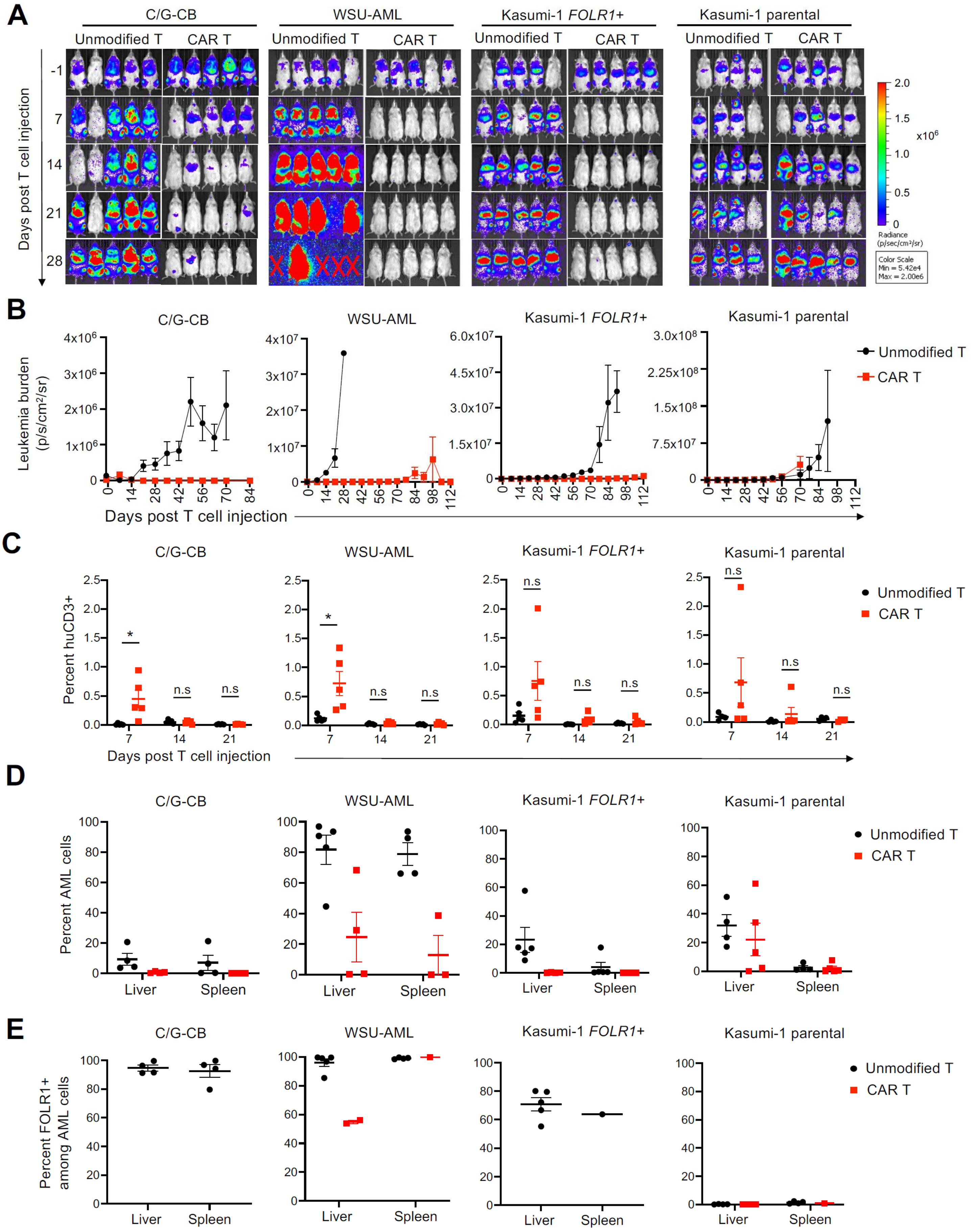
A
C/G-CB
WSU-AML
Kasumi-1 FOLR1+
Kasumi-1 parental
Unmodified T
CAR T
Days post T cell injection
-1
7
14
21
28
Radiance (p/sec/cm²/sr)
Color Scale Min = 5.42e4 Max = 2.00e6
B
Leukemia burden (p/s/cm²/sr)
Days post T cell injection
Unmodified T
CAR T
C
Percent huCD3+
n.s
*
D
Percent AML cells
Liver
Spleen
E
Percent FOLR1+ among AML cells

Supplemental Figure 12. In vivo efficacy of FOLR1-directed CAR T. (Related to Figure 6) A. Bioluminescent imaging of C/G-CB, WSU-AML, Kasumi-1 *FOLR1*+ and Kasumi-1 leukemias in mice treated with unmodified or FOLR1 CAR T cells at 5 $x10^6$ T cells per mouse. Shown are the images one day before T cell treatment and 4 weeks after T cell treatment. N=5 mice/ group. Radiance scale indicates an increase in leukemia from blue to red; X indicates death. **B.** Quantification of leukemia burden over time based on IVIS radiance in CBFA2T3-GLIS2-transduced HSPCs, WSU-AML, Kasumi-1 FOLR1+ and Kasumi-1 xenografts treated with unmodified or FOLR1 CAR T cells at 5 $x10^6$/mouse. Leukemia burden is shown for each mouse. N = 5 mice per group. Data presented are the average +/- standard deviation from 5 mice. *, $p<0.05$ (unpaired Student's t-test). **C.** Quantification of human T cells in the mouse peripheral blood at indicated time points after T cell injection. Shown is human CD45+CD3+ frequency amongst DAPI- cells. N = 5 mice per group. Data presented are the average +/- standard deviation from 5 mice. *, $p<0.05$ (unpaired Student's t-test). **D, E.** Quantification of percent leukemia cells (human CD45+, D) and percent FOLR1 positive amongst leukemia cells (E) in the liver and spleen tissues at necropsy (either when mice developed symptomatic leukemia or euthanized at the indicated study endpoint. Note that some mice are missing because they were found dead in cage (FDIC), or tissues were not viable. For cases where leukemia burden is absent, FOLR1 expression could not be determined for these mice and were excluded from the analysis. Data presented are the average +/- standard deviation *, $p<0.05$ (unpaired Student's t-test).

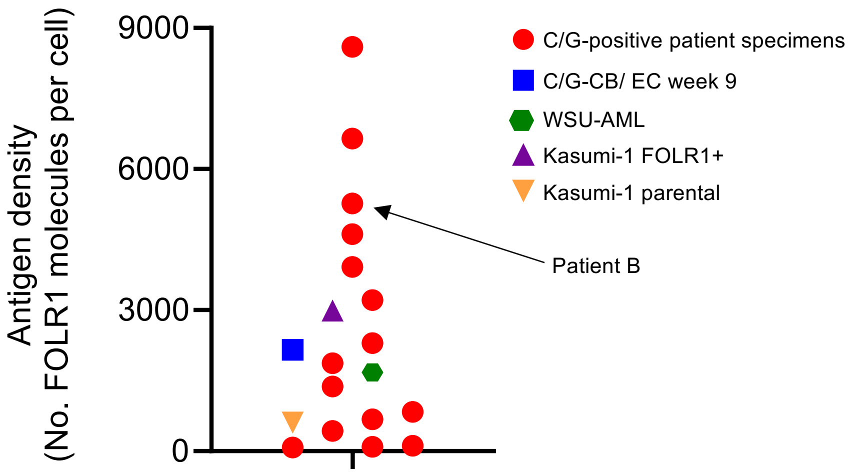

Supplemental Figure 13. FORL1 cell surface expression across patient samples, C/G-CB cells, cell lines and engineered cells. Flow analysis of antigen density (number of FOLR1 molecules per cell) across C/G-positive patient specimens, C/G-CB cells after 9 weeks in EC co-culture, WSU-AML, Kasumi-1 FOLR1+ and Kasumi-1 parental cells. Arrow points to C/G-positive patient (Patient B) used in the evaluation of FOLR1 CAR T cells (Figure 7).

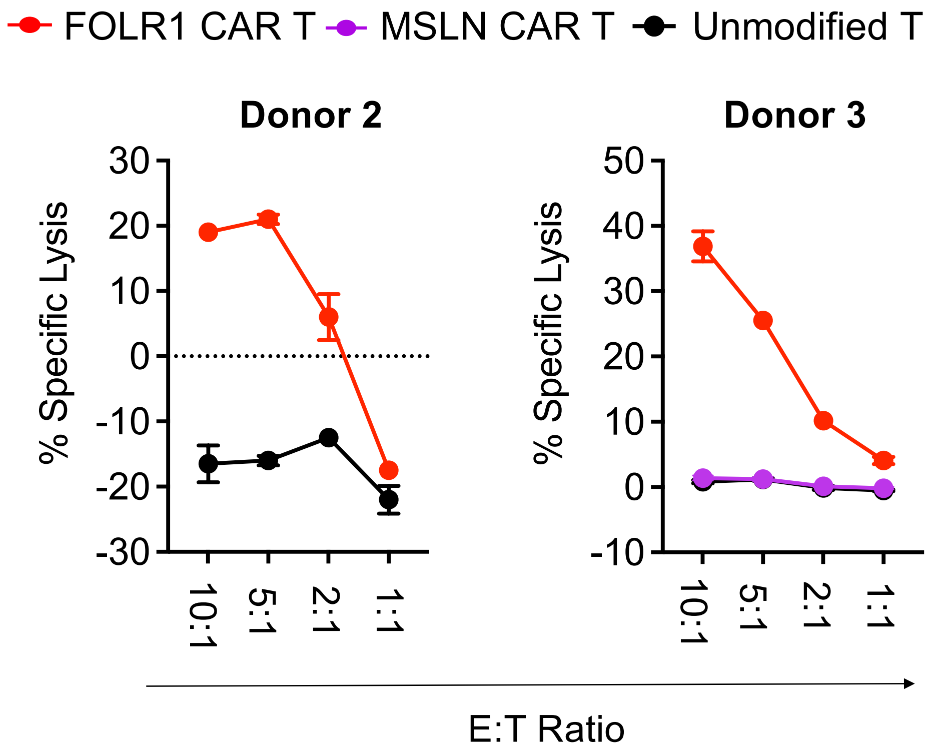

Supplemental Figure 14. In vitro cytotoxicity of FOLR1 CAR T cells against primary C/G AML cells (Related to Figure 7) Cytolytic activity of CD8 T cells unmodified or transduced with FOLR1 CAR or MSLN CAR following 6 hours of co-culture with primary C/G AML cells. CAR T cells were produced from two additional donors (separate from the donor shown in Figure 7).

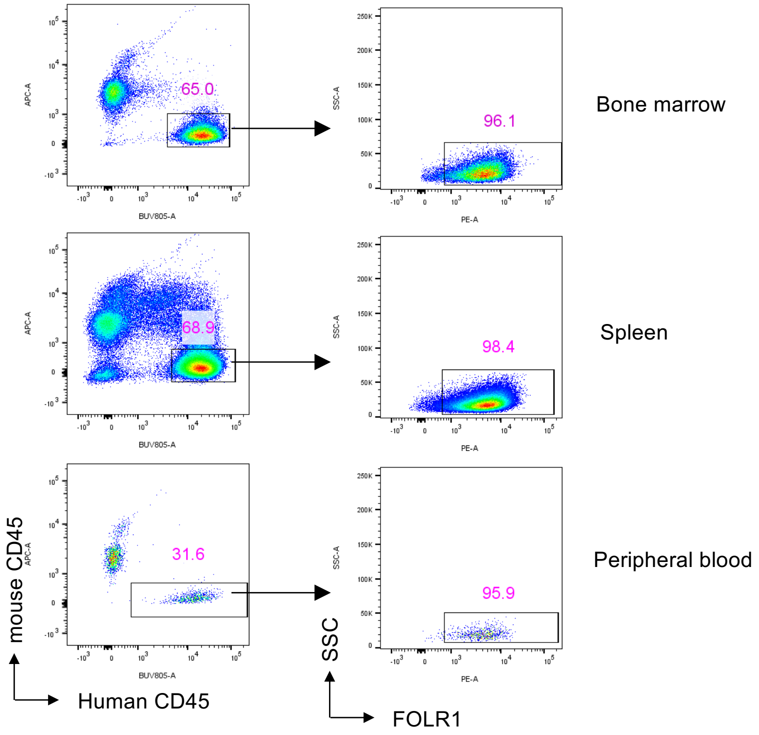

Supplemental Figure 15. Expression of FOLR1 in primary AML cells that were present in CAR T-treated mouse that developed symptomatic leukemia at 180 days post T cell injection. (Related to Figure 7) Left, percent of human CD45+ cells in the bone marrow, spleen and blood of the CAR T-treated mouse that relapsed at day 180 post T cell injection. Right, percent of human CD45+ cells that expression FOLR1.

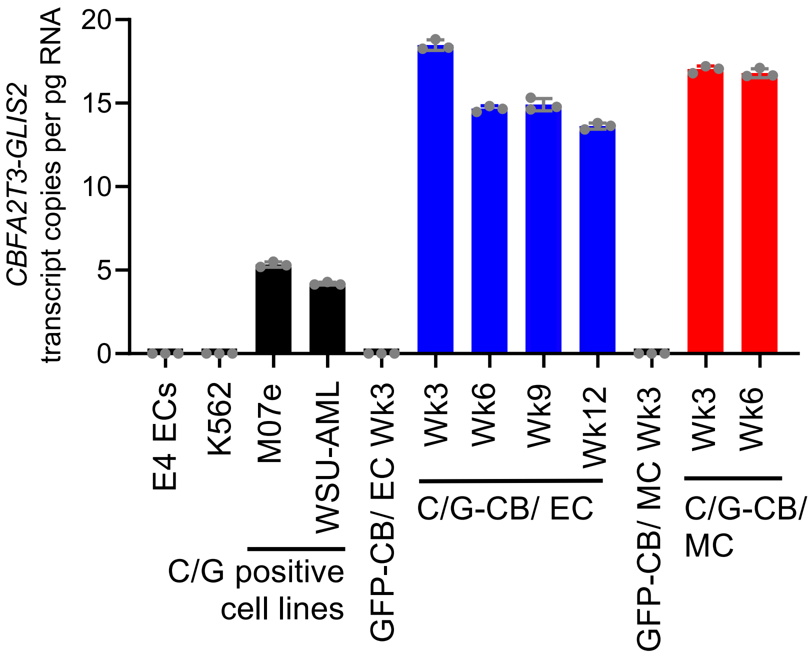

Supplemental Figure 16. Expression of *C/G* transcript in C/G-CB cells. RT-PCR analysis of *C/G* expression in engineered CB cells and in fusion positive cell lines M07e and WSU-AML. Data shown is the average of 3 technical replicates. Error bars denote standard deviation.

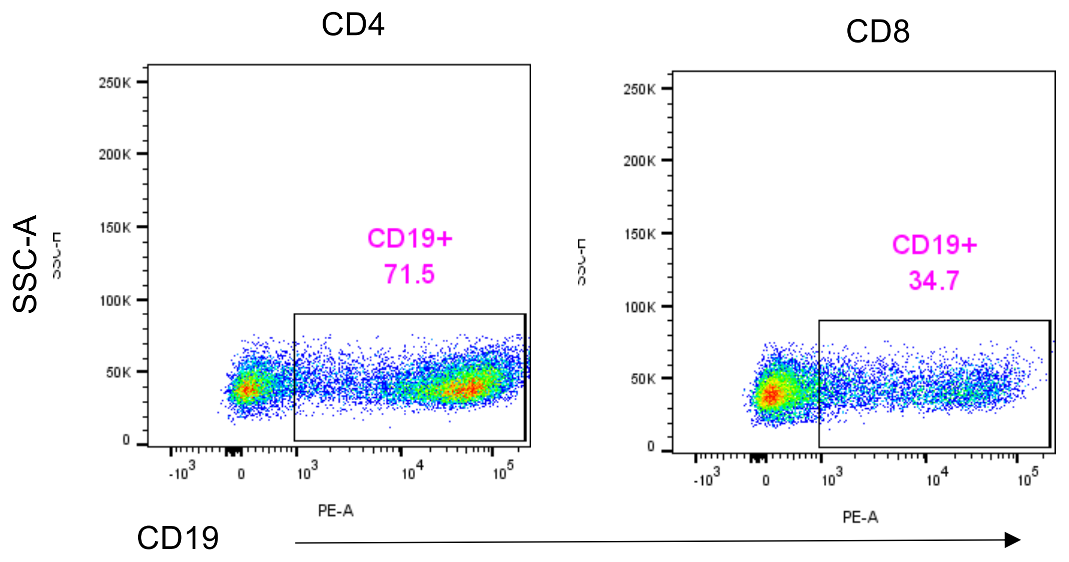

Supplemental Figure 17. Transduction efficiency of FOLR1 CAR construct. Representative transduction efficiency of CD4 and CD8 T cells with FOLR1 CAR (intermediate spacer) lentivirus after 7 days post transduction.

Supplemental Information

Constructs and Lentivirus production

The MSCV-CBFA2T3-GLIS2-IRES-mCherry construct was a gift from Dr. Tanja Gruber (Department of Oncology, St. Jude Children's Research Hospital, Memphis, Tn, Ref(1)). The *C/G* fusion gene from this construct and the MND promoter were inserted into pRRLhPGK-GFP lentivirus vector(2) as described in Ref.(3).

CAR constructs containing IgG4 short, intermediate and long spacers are previously described in Ref.(4). The VL and VH sequences from Farletuzumab were used to construct the anti-FOLR1 scFv with VL/VH orientation using G4SX4 linker. Anti-FOLR1 scFv DNA fragment was human codon optimized and synthesized by IDT gBlock gene fragment and cloned into the CAR vectors with NheI and RsrII restriction sites upstream of the IgG4 spacer. MSLN CAR construct with short spacer used in this study as an irrelevant CAR control was previously described Ref. (37).

Lentivirus particles were produced in 293T cells (ATCC, Cat#CRL-3216). 293T cells were transfected with transfer vector, viral packaging vector (psPAX2), and viral envelope vector (pMD2G) at 4:2:1 ratio using Mirus 293Trans-IT transfection agent (Mirus, Cat# MIR2700) as directed by manufacturer's protocol. Viral particles were collected each day for 4 days post transfection, filtered through 0.45 µm membrane (Thermo Fisher; Cat NAL-166-0045) and concentrated (overnight spin at 4ºC, 5000rpm) before use.

Farletuzumab scFv

DIQLTQSPSSLSASVGDRVTITCSVSSSISSNNLHWYQQKPGKAPKPWIYGTSNLASGVPSRFS
GSGSGTDYTFTISSLQPEDIATYYCQQWSSYPYMYTFGQGTKVEIKGGGGSGGGGSGGGGS

GGGGSEVQLVESGGGVVQPGRSLRLSCSASGFTFSGYGLSWVRQAPGKGLEWVAMISSGGS YTYYADSVKGRFAISRDNAKNTLFLQMDSLRPEDTGVYFCARHGDDPAWFAYWGQGTPVTVS S

RNA-seq analysis

Screening of C/G Fusion in patient samples. The *C/G* fusion transcript was detected by Fragment length analysis or fusion detection algorithms STAR-fusion v1.1.0 and TransAbyss v1.4.10(5, 6). Details of the procedure are described previously(3).

Transcriptome Analysis: Differentially expressed genes between C/G-CB and GFP-CB cells were identified using the limma voom (v3.44.3 R package) with trimmed mean of M values (TMM) normalized gene counts(7). Genes with absolute log2 fold-change > 1 and Benjamini–Hochberg adjusted p-values < 0.05 were retained. Unsupervised hierarchical clustering was completed using the ComplexHeatmap R package (v2.4.3), utilizing Euclidean distances with the ward.D2 linkage algorithm. Log2 transformed TMM normalized counts per million (CPM) were used as input, with a count of 1 added to avoid taking the log of zero. Hierarchical clustering of primary C/G AML samples and C/G-CB cells using a C/G transcriptome signature was carried out. The signature genes (N=1,116 genes) were defined as those within the 75th percentile of absolute log2 fold-changes and adj. p.value < 0.001, when contrasting C/G fusion positive patients (N=39) against a heterogenous AML reference cohort (N=1,355). The 85th percentile of this signature (N = 167 genes) was used to define a C/G gene set in GSEA.

Gene-set enrichment scores were calculated using the single-sample gene-set enrichment (ssGSEA) method (GSVA v1.32.0), which transforms normalized count data from a gene by sample matrix to a gene-set by sample matrix(8). Counts were TMM normalized and log2(x+1) transformed prior to gene-set analysis. Curated signaling and metabolic gene-sets

from the KEGG database were included in the analysis (gageData v2.26.0). Significant gene-sets (Benjamini–Hochberg adjusted p-values < 0.05) associated with C/G-CB cells were identified using limma v3.44.3 with the GSVA transformed gene-set by sample matrix as input.

GSEA was performed using the 'unpaired' comparison in the GAGE R package (v2.38.3), which tests for differential expression of gene-sets by contrasting C/G-CB against GFP-CB cells in each condition to define pathways enriched in EC co-culture versus MC. Non-redundant gene-sets were extracted for further analysis, followed by the identification of core genes that contribute to the pathway enrichment. Gene-sets from the Molecular Signatures Database (MSigDB) and the KEGG pathway database were used. Enrichment score plots for the HSC and C/G signatures were generated using the R package fgsea (v1.14.0). Log fold change values obtained from limma (contrasting C/G-CB EC week 6 against C/G-CB MC week 6) were used as a ranking metric for genes in the two signatures.

Unsupervised clustering of C/G-CB cells with pediatric AML primary diagnostic samples (N=1,033) and healthy normal bone marrows (N=68) was performed by uniform manifold approximation and projection (UMAP) using the uwot v0.1.8 R package(9). For UMAP clustering, gene counts underwent variance stabilizing transformation (VST) using the DESeq2 v1.28.1 package. Input genes for clustering (N=6,678 genes) were selected using the mean versus dispersion parametric model trend (SeqGlue v0.1) to identify genes with high variability.

Identification of fusion-specific CAR targets involves three main steps: 1) Determine the ratio of expression for AML primary samples versus healthy normal hematopoietic tissue samples (bulk normal bone marrow, N=68, in combination with CD34+ selected peripheral blood samples, N=16) from log10 transformed normalized expression as transcripts per million, (TPM). Normalization was completed on the full gene expression matrix followed by ratio analysis on 19,901 annotated protein-coding genes for the identification of therapeutic targets. The ratio is calculated per gene from the mean expression in AML and normal tissues, where normal healthy hematopoietic tissue mean expression is the divisor, which acts as a measure of

over or under expression. A normal curve is fit to the ratio values, and genes with ratios greater than +2 standard deviations were retained. This process is carried out for all heterogenous AML samples (N=1483) as a group and then repeated iteratively within AML fusion and mutation subtypes, including C/G, to ensure the inherent variability of gene expression in different fusion classes is addressed and all viable targets are identified for any given subtype. Genes are then further refined to include those with maximum expression < 1.0 TPM in normal healthy hematopoietic tissue samples, and thus considered to have AML restricted expression when compared to healthy controls. 2) AML restricted genes were further selected if found to be significantly overexpressed by RNA-seq for bulk fusion positive patient samples compared to bulk healthy bone marrows and were likewise overexpressed in C/G-CB at weeks 6 and 12 in EC co-culture with an absence of expression (< 1.0 TPM) in GFP-CB controls providing several candidate targets. 3). Final selection of optimal CAR-T targets was determined by the identification of candidate genes with cell surface localization potential as annotated by the Human Protein Atlas (https://www.proteinatlas.org/) or Jensen Lab compartments database (https://compartments.jensenlab.org/), in addition to having moderate to high expression in C/G patient samples (maximum expression ≥ 10 TPM), expression in a majority (> 75%) of patient samples, and an absence of expression in healthy hematopoietic tissues as noted in step 1 above.

Cell surface analysis

For xenograft CB cells, mouse bone marrow, peripheral blood, spleen, and liver were harvested at necropsy and processed with red blood cell lysis buffer. Spleen and liver were processed into cell suspension with glass slides and passed through a 70-um cell strainer. CB cells in EC co-culture and MC were harvested after vigorously pipetting to resuspend CB cells. CB cells from processed mouse tissues and cultures were washed in 2% FBS in PBS, blocked with 2% human AB serum in PBS, then stained with a cocktail of fluorescently labeled

monoclonal antibodies for 20 min on ice (see Supplemental Information for antibodies used). Labeled cells were washed with PBS and resuspended in 2% FBS/PBS prior to flow cytometric analysis. FACSymphony equipped with FACSDiva Software (BD Biosciences) was used to assess cell surface expressions and FlowJo Software was used for the analysis. Dead cells were excluded based on LIVE/DEAD^{TM} Fixable Violet Dead Cell Stain (FVD, Invitrogen, cat# L34955). For EC co-cultures, ECs were excluded by gating on CD45+ cells or CD45+CD144- cells.

A fraction of the C/G-CB cells isolated from xenograft models or cultured in EC co-culture or MC at various timepoints were sent to Hematologics, Inc (Seattle, WA) for assessment of the RAM immunophenotype along with C/G patient samples.

Antibody Cocktails

Antibody panel to assess transduced CB cells in xenograft mice:

- anti-mouse CD45.1 PE, cat#553776 (clone A20) BD Biosciences
- anti-human CD45 BUV805, cat#612891 (clone HI30) BD Biosciences
- anti-human CD3 BUV395, cat#564001 (clone SK7) BD Biosciences
- anti-human CD19 APC-eFluor780, cat#47-0198-42 (clone SJ25C1) eBioscience
- anti-human CD33 APC, cat#340474 (clone P67.6) BD Biosciences
- anti-human CD34 PE-Cy7, cat#348801 (clone 8G12) BD Biosciences
- anti-human CD41a BV650, cat#740575 (clone HIP8) BD Biosciences
- anti-human CD42b BUV661, cat#741614 (clone HIP1) BD Biosciences
- anti-human CD56 BUV737, cat#748609 (clone MY31) BD Biosciences
- anti-human CD117 PerCP-Cy5.5, cat#313214 (clone 104D2) Biolegend

Antibody panel to assess cultured CB cells:

- anti-human CD14 APC-Cy7, cat#557831 (clone MϕP-9) BD Biosciences
- anti-human CD15 BUV805, cat#742057 (clone W6D3) BD Biosciences
- anti-human CD33 BUV395, cat#745709 (clone 104D2) BD Biosciences
- anti-human CD34 PE-Cy7, cat#348801 (clone P67.6) BD Biosciences
- anti-human CD41a BV650, cat#740575 (clone HIP8) BD Biosciences
- anti-human CD42b BUV661, cat#741614 (clone HIP1) BD Biosciences
- anti-human CD45 APC, cat#17-9459-42 (clone 2D1) eBioscience
- anti-human CD56 BUV737, cat#748609 (clone MY31) BD Biosciences
- anti-human FOLR1 PE, cat#908304 (clone LK26) Biolegend
- anti-human FOLR2 PE, cat#391703 (clone 94b) Biolegend

<u>Antibody panel to assess unmodified/ CAR T and leukemia cells in xenograft mice:</u>

- anti-mouse CD45.1 APC/Cy7, cat#110716 (clone A20) Biolegend
- anti-human CD45 BUV805, cat#612891 (clone HI30) BD Biosciences
- anti-human CD3 PE-Cy7, cat#563423 (clone UCHT1) BD Biosciences
- anti-human FOLR1 PE, cat#908304 (clone LK26) Biolegend

<u>Antibody panel to assess FOLR1 expression in normal HSC, MPP and MLP subsets:</u>

- anti-human CD34 BUV395 cat#563778 (clone 581) BD Biosciences
- anti-human CD38 PE-Cyanine7, cat#25-0389-42 (clone HIT2) eBioscience
- anti-human CD45 BUV805, cat#612891 (clone HI30) BD Biosciences
- anti-human CD45RA APC-eFluor780, cat#47-0458-42 (clone HI100) eBioscience
- anti-human CD90 PE-Cy5, cat#555597 (clone 5E10) BD Bioscience
- anti-human FOLR1 PE, cat#908304 (clone LK26) Biolegend

Antibody panel to assess FOLR1 expression in normal CLP, GMP, CMP and MEP subsets:

- anti-human CD10 B605, cat#562978 (clone HI10a) BD Biosciences
- anti-human CD34 BUV395 cat#563778 (clone 581) BD Biosciences
- anti-human CD38 PE-Cyanine7, cat# 25-0389-42 (clone HIT2) eBioscience
- anti-human CD45 BUV805, cat# 612891 (clone HI30) BD Biosciences
- anti-human CD45RA APC-eFluor780, cat# 47-0458-42 (clone HI100) eBioscience
- anti-human CD123 APC cat#658171 (clone 9F5) BD Biosciences
- anti-human FOLR1 PE, cat#908304 (clone LK26) Biolegend

Colony-forming cell assay

Following 6 and 12 weeks of culture, cells were placed in or Megacult (Megacult-C, Collagen & Medium with Cytokines Stemcell Technologies, Cat #04961) and incubated at 37°C in 5% CO_2 for 10-14 days. Colonies from megacult cultures were fixed in 3.7% formaldehyde, and then washed in PBS, and stained with MegaCult™-C Staining Kit for CFU-Mk (StemCell Technologies Cat# 04962) per manufacturer's instructions; or were permeabilized after fixation in 0.1% Triton X-100 for 10min, blocked in in 1% BSA in PBST(PBS+0.1% Tween-20) for 30min, then stained with biotin-conjugated mouse anti-human CD41 (Biolegend, cat# 303734) and FITC-conjugated goat anti-GFP(abcam, cat# ab6662) followed by secondary stain with Alexa 647-labeled Streptavidin (Biolegend, cat# 405237) per manufacturer's instructions, and colonies were stained with DAPI prior to imaging using the TissueFAX microscope. Mk colonies were scored based on positive staining for CD41 and enumerated.

C/G-CB and normal HPSCs after co-cultured with unmodified or CAR T cells for 4 hours were placed in Methocult H4034 Optimum (Stemcell Technologies, Cat #04034). Colonies derived from erythroid (E), granulocyte-macrophage (G, M, and GM) and multipotential

granulocyte, erythroid, macrophage, megakaryocyte (GEMM) progenitors were scored and enumerated after 7-10 days as directed by manufacturer's instructions.

Histology and immunocytochemistry

Sample tissues were fixed in 10% formalin, processed into paraffin sections and stained with hematoxylin and eosin (H&E). Immunohistochemistry was performed using antibodies to ERG (EP111; Cell Marque, Rockin, California) and CD56 (MRQ-42; Cell Marque) following citrate pretreatment and visualized with 3, 3'-diaminobenzidine (DAB) on a Ventana Bench Mark Ultra. All tissues were examined by a board certified Hematopathologist (KRL). The bone marrow core biopsy specimen was fixed in acetic acid-zinc-formalin (AZF), decalcified, and embedded in paraffin, and sections were stained for CD56 (MRQ-42; Cell Marque).

Tissue microarray (TMA) slides were purchased from US Biomax; sectioned at 5μm and stored at 4°C. Prior to staining, slides were baked at 60°C for 2 hours to ensure tissue adhesion. Staining was completed using a Ventana Discovery Ultra platform and corresponding reagents. After deparaffinization, antigen retrieval was performed using cell conditioner 1 (CC1, Cat# 950-224) at 95°C for 32mins. Blocking of endogenous background was done using Inhibitor CM (ChromoMap DAB Kit; cat# 760-159) for 12mins. The primary antibody (FolR1a, 26B3.F2; Morphotek), at a concentration of 1:300 in antibody diluent (Roche, Cat# 760-219), was incubated at 35°C for 28mins after which a secondary antibody/hapten linked solution (Anti-Mouse HQ, Cat# 760-4814) was introduced at 37°C for 12mins. Slides were then immersed in a hapten/HRP linked conjugate (Anti-HQ-HRP, Cat# 3 760-4820) for a further 12mins before developing using DAB (ChromoMap DAB). After chromogen attachment, the slides are counterstained with hematoxylin (Cat# 760-2021) and bluing solution (Cat# 760-2037) at 18°C for 8 mins each. Slides are then washed in soap water solution (Dawn), rinsed in DI water, dehydrated, cleared in xylene and mounted (Permount; Fisher Scientific, Cat# 5027798) for

microscopic evaluation. Slides were scanned on the Aperio AT Turbo (Leica, Deer Park, IL) and analyzed using the HALO analysis software (Indica Labs, v3.1, Albuquerque, NM)

References for Supplemental Information

1. Gruber TA, Larson Gedman A, Zhang J, Koss CS, Marada S, Ta HQ, et al. An Inv(16)(p13.3q24.3)-encoded CBFA2T3-GLIS2 fusion protein defines an aggressive subtype of pediatric acute megakaryoblastic leukemia. *Cancer Cell.* 2012;22(5):683-97.
2. Dull T, Zufferey R, Kelly M, Mandel RJ, Nguyen M, Trono D, et al. A third-generation lentivirus vector with a conditional packaging system. *J Virol.* 1998;72(11):8463-71.
3. Smith JL, Ries RE, Hylkema T, Alonzo TA, Gerbing RB, Santaguida MT, et al. Comprehensive Transcriptome Profiling of Cryptic CBFA2T3-GLIS2 Fusion-Positive AML Defines Novel Therapeutic Options: A COG and TARGET Pediatric AML Study. *Clin Cancer Res.* 2020;26(3):726-37.
4. Turtle CJ, Hanafi LA, Berger C, Hudecek M, Pender B, Robinson E, et al. Immunotherapy of non-Hodgkin's lymphoma with a defined ratio of CD8+ and CD4+ CD19-specific chimeric antigen receptor-modified T cells. *Sci Transl Med.* 2016;8(355):355ra116.
5. Haas BJ, Dobin A, Li B, Stransky N, Pochet N, and Regev A. Accuracy assessment of fusion transcript detection via read-mapping and de novo fusion transcript assembly-based methods. *Genome Biol.* 2019;20(1):213.
6. Robertson G, Schein J, Chiu R, Corbett R, Field M, Jackman SD, et al. De novo assembly and analysis of RNA-seq data. *Nat Methods.* 2010;7(11):909-12.
7. Ritchie ME, Phipson B, Wu D, Hu Y, Law CW, Shi W, et al. limma powers differential expression analyses for RNA-sequencing and microarray studies. *Nucleic Acids Res.* 2015;43(7):e47.
8. Hanzelmann S, Castelo R, and Guinney J. GSVA: gene set variation analysis for microarray and RNA-seq data. *BMC Bioinformatics.* 2013;14:7.
9. Leland McInnes JH, James Melville. UMAP: Uniform ManifoldApproximation and Projection forDimension Reduction. *arxiv.* 2020:

.

Supplemental Figure Legends

Supplemental Figure 1. C/G-CB cells form tight clusters in mouse bone marrow. (Related to Figure 1) H&E stains of femurs taken from primary, secondary and tertiary transplants of C/G-CB cells. Magnification: 2.5X(left), 10X (middle), 40X (right).

Supplemental Figure 2. Expression of CD56 and AMKL markers in C/G-CB xenograft cells following development of symptomatic leukemia in NSG-SGM3 mice. (Related to Figure 1) A. Percent human $CD45^+$ cells in the bone marrow, spleen, liver and peripheral blood (PB) from mice transplanted with C/G-CB cells in primary (1°, N=4 mice), secondary (2°, N=7 mice) and tertiary (3°, N=5 mice) transplants. **B, C.** Percent $CD56^+$ and CD41/CD42 subsets in mouse tissues described in A. 1°, N=4 mice per group; 2°, N=7 mice; and 3°, N=5 mice.

Supplemental Figure 3. Assessment of RAM and AMKL markers in C/G-CB cells isolated from mice transplanted with engineered cells cultured in EC co-culture or MC. (Related to Figure 2) A. Percent human CD45+ cells in the bone marrow, spleen liver and peripheral blood from mice transplanted with C/G-CB and GFP-CB cells at indicated timepoints in EC co-culture or MC. **B, C.** Percent CD41/CD42 subsets (B) andCD56+ cells (C) among live human CD45+ in mouse tissues described in A. Data analyzing CB cells in the liver for mice transplanted with GFP-CB cells from MC culture are not included as not enough cells were presence in these samples. Same goes for peripheral blood data from 2 mice transplanted with C/G-CB cells grown in MC. C/G-CB (N=3 mice/group), GFP-CB (N=2 mice/group)

Supplemental Figure 4. ECs promote transformation of C/G-CB cells. (Related to Figure 2) A. Schematic of transduction and long-term cultures of cord blood CD34+ HSPCs from a second donor. **B.** Growth kinetics of transduced cells over days in EC or MC as determined by the cumulative number of GFP+ cells. Mean +/- standard deviation from 3 technical replicates

are shown. Growth rate constant k was determined by regression analysis using the formula $N(t) = N(0)e^{kt}$ where *t* is measured in days. **C.** Following 6 and 12 weeks of culture, a fraction of each culture was transferred to megacult to enumerate Mk colonies. Data are normalized to the CD34+ input cells at the start of the culture and presented as mean +/- standard deviation from 3 technical replicates. **D.** Expression of the RAM immunophenotype in C/G-CB cells after 6 weeks in either EC co-culture or MC.

Supplemental Figure 5. Expression of C/G-specific genes. (Related to Figure 3) Heat maps showing expression of C/G-specific focal adhesion and cell adhesion molecule genes (**A**), genes associated with primary C/G fusion positive AML (**B**), and HSC signature genes (**C**). Unsupervised hierarchical clustering demonstrates clustering of C/G-CB cells cultured with ECs for 6 and 12 weeks with primary C/G samples. CBFA2T3-GLIS2 primary samples (N=39); NBM samples (N=68). For cultured cells, N=4 technical replicates for C/G-CB cells in EC co-culture at week; N=3 technical replicates for all other groups.

Supplemental Figure 6. Identification of C/G fusion-specific CAR targets. (Related to Figure 4A) Flow diagram of AML-restricted gene and CAR-T target identification. The procedure involves three main steps: 1) Determine the ratio of expression for AML primary samples versus healthy normal hematopoietic tissue samples (bulk marrows and CD34+ peripheral blood) from log10 transformed normalized gene expression. The ratio is calculated per gene from mean AML expression and mean normal hematopoietic tissue expression, where normal tissue values are the divisor, which acts as a measure of over or under expression. A normal curve is fit to the ratios and this procedure is completed for all heterogenous AML samples as a group, and iteratively within fusion and mutation subtypes; genes with ratios greater than +2 standard deviations and with absent expression in normal hematopoietic tissues were retained (N=607) for further analysis. 2) The AML restricted genes were further selected if

found to be significantly overexpressed in fusion positive patient samples compared to healthy marrows and were likewise overexpressed in C/G-CB at weeks 6 and 12 in EC co-culture with absent expression in GFP-CB controls, providing several candidate (N=42) targets. 3) Optimal CAR-T targets were selected by the identification of candidate genes with cell surface localization potential, and those with an absence of expression in healthy tissue controls as noted in step 1, but expression in > 75% of C/G patient samples, and with moderate to high expression levels (N=6).

Supplemental Figure 7. *FOLR1* transcript is uniquely expressed in C/G AML. Waterplot showing transcript expression (TPM) among C/G AML (red, N=39), other AML (pink, N=1,453), peripheral blood (PB) CD34+ samples (grey, N=16) and bulk bone marrow samples (black, N=68).

Supplemental Figure 8. Expression of *FOLR1* transcript in C/G-CB cells cultured on ECs. RT-PCR analysis of *FOLR1* expression in engineered CB cells and in fusion positive cell lines M07e and WSU-AML. Expression is normalized as fold-change relative to GFP-CB/EC Wk 3 samples. Data shown is the average of 3 technical replicates. Error bars denote standard deviation.

Supplemental Figure 9. Expression of FOLR2 (folate receptor beta) in C/G AML. A. Heat map showing expression of FOLR1 and FOLR2 among C/G-positive patients at diagnostic and relapse, and in bone marrow samples from healthy donors. **B, C.** Transcript (B) and cell surface (C) expression of FORL1 and FOLR2 in C/G-CB and GFP-CB cells over weeks in EC co-culture.

Supplemental Figure 10. Expression of FOLR1 in normal tissues. Immunohistochemistry of

FOLR1 staining in 32 types of normal human organs (Tissue Microarray, TMA FDA999u), each type taken from 3 different individuals. Top, sample ID; bottom, FOLR1-stained TMA.

Supplemental Figure 11. In vitro reactivity of FOLR1 CAR T cells against C/G AML cells. **A.** Flow cytometric analysis of FOLR1 and MSLN expression in C/G-CB cells and WSU-AML cell line. **B.** Cytolytic activity of CD8 T cells unmodified or transduced with FOLR1 CAR or MSLN CAR following 6 hours of co-culture with C/G-CB (cell taken from >9 weeks in EC co-culture) or WSU-AML. Data presented are mean leukemia specific lysis +/- SD from 3 technical replicates at indicated effector: target (E:T) ratios. T cells were derived from two additional donors. **C.** Concentration of secreted IL-2, IFN-**γ**, and TNF-**α** in the supernatant following 24 hour of T cell/AML co-culture at 1:1 E:T ratio as measured by ELISA. Data are derived from T cells isolated from donor 2 and are presented as mean +/- SD from 3 technical replicates. Where concentrations of cytokines are too low to discern, the number above the x-axis indicates the average concentration. **D.** Left, flow cytometric analysis of FOLR1 and MSLN expression in Nomo-1 AML cell line. Middle, cytolytic activity of CD8 T cells unmodified or transduced with FOLR1 CAR or MSLN CAR following 24 hours of co-culture with Nomo-1 cells. Data presented are mean leukemia specific lysis +/- SD from 3 technical replicates at indicated E:T ratios. Right, concentration of secreted IL-2, IFN-**γ**, and TNF-**α** in the supernatant following 24 hour of T cell/AML co-culture at 1:1 E:T ratio as measured by ELISA. Data are derived from T cells isolated from donor 2. **E.** Flow cytometric analysis of FORL1 in WSU-AML FOLR1 knock out (KO). **F.** Cytolytic activity of CD8 T cells unmodified or transduced with FOLR1 CAR or MSLN CAR following 24 hours of co-culture with WSU-AML cells or WSU-AML FOLR1 KO cells. Data are derived from T cells isolated from donor 2 and are presented as mean +/- SD from 3 technical replicates. **G.** Concentration of secreted IL-2, IFN-**γ**, and TNF-**α** in the supernatant following 24 hour of T cell/AML co-culture at 1:1 E:T ratio as measured by ELISA. Data are derived from T cells isolated from donor 2 and are presented as mean +/- SD from 3 technical

replicates. Where concentrations of cytokines are too low to discern, the number above the x-axis indicates the average concentration.

Supplemental Figure 12. In vivo efficacy of FOLR1-directed CAR T. (Related to Figure 6) **A.** Bioluminescent imaging of C/G-CB, WSU-AML, Kasumi-1 *FOLR1+* and Kasumi-1 leukemias in mice treated with unmodified or FOLR1 CAR T cells at 5 $x10^6$ T cells per mouse. Shown are the images one day before T cell treatment and 4 weeks after T cell treatment. N=5 mice/ group. Radiance scale indicates an increase in leukemia from blue to red; X indicates death. **B.** Quantification of leukemia burden over time based on IVIS radiance in CBFA2T3-GLIS2-transduced HSPCs, WSU-AML, Kasumi-1 FOLR1+ and Kasumi-1 xenografts treated with unmodified or FOLR1 CAR T cells at 5 $x10^6$/mouse. Leukemia burden is shown for each mouse. N = 5 mice per group. Data presented are the average +/- standard deviation from 5 mice. *, $p<0.05$ (unpaired Student's t-test). **C.** Quantification of human T cells in the mouse peripheral blood at indicated time points after T cell injection. Shown is human CD45+CD3+ frequency amongst DAPI- cells. N = 5 mice per group. Data presented are the average +/- standard deviation from 5 mice. *, $p<0.05$ (unpaired Student's t-test). **D, E.** Quantification of percent leukemia cells (human CD45+, D) and percent FOLR1 positive amongst leukemia cells (E) in the liver and spleen tissues at necropsy (either when mice developed symptomatic leukemia or euthanized at the indicated study endpoint. Note that some mice are missing because they were found dead in cage (FDIC), or tissues were not viable. For cases where leukemia burden is absent, FOLR1 expression could not be determined for these mice and were excluded from the analysis. Data presented are the average +/- standard deviation *, $p<0.05$ (unpaired Student's t-test).

Supplemental Figure 13. FORL1 cell surface expression across patient samples, C/G-CB cells, cell lines and engineered cells. Flow analysis of antigen density (number of FOLR1

molecules per cell) across C/G-positive patient specimens, C/G-CB cells after 9 weeks in EC co-culture, WSU-AML, Kasumi-1 FOLR1+ and Kasumi-1 parental cells. Arrow points to C/G-positive patient (Patient B) used in the evaluation of FOLR1 CAR T cells (Figure 7).

Supplemental Figure 14. In vitro cytotoxicity of FOLR1 CAR T cells against primary C/G AML cells (Related to Figure 7) Cytolytic activity of CD8 T cells unmodified or transduced with FOLR1 CAR or MSLN CAR following 6 hours of co-culture with primary C/G AML cells. CAR T cells were produced from two additional donors (separate from the donor shown in Figure 7).

Supplemental Figure 15. Expression of FOLR1 in primary AML cells that were present in CAR T-treated mouse that developed symptomatic leukemia at 180 days post T cell injection. (Related to Figure 7) Left, percent of human CD45+ cells in the bone marrow, spleen and blood of the CAR T-treated mouse that relapsed at day 180 post T cell injection. Right, percent of human CD45+ cells that expression FOLR1.

Supplemental Figure 16. Expression of *C/G* transcript in C/G-CB cells. RT-PCR analysis of *C/G* expression in engineered CB cells and in fusion positive cell lines M07e and WSU-AML. Data shown is the average of 3 technical replicates. Error bars denote standard deviation.

Supplemental Figure 17. Transduction efficiency of FOLR1 CAR construct. Representative transduction efficiency of CD4 and CD8 T cells with FOLR1 CAR (intermediate spacer) lentivirus after 7 days post transduction.
